# Function of cytochrome P450 *CYP72A1182* in metabolic herbicide resistance evolution in *Amaranthus palmeri* populations

**DOI:** 10.1093/jxb/eraf114

**Published:** 2025-03-11

**Authors:** Carlos Alberto Gonsiorkiewicz Rigon, Anita Küpper, Crystal Sparks, Jacob Montgomery, Falco Peter, Simon Schepp, Alejandro Perez-Jones, Patrick J Tranel, Roland Beffa, Franck E Dayan, Todd A Gaines

**Affiliations:** Colorado State University, Department of Agricultural Biology, Fort Collins, CO 80523, USA; Bayer AG, Division CropScience, Weed Control Research, 65926 Frankfurt, Germany; Colorado State University, Department of Agricultural Biology, Fort Collins, CO 80523, USA; Colorado State University, Department of Agricultural Biology, Fort Collins, CO 80523, USA; Bayer AG, Division CropScience, Weed Control Research, 65926 Frankfurt, Germany; Bayer AG, Division CropScience, Weed Control Research, 65926 Frankfurt, Germany; Weed Control Platform Lead, Bayer CropScience, St Louis, MO 63141, USA; University of Illinois, Department of Crop Sciences, Urbana, IL 61801, USA; Senior Scientist Consultant, 65835, Liederbach, Germany; Colorado State University, Department of Agricultural Biology, Fort Collins, CO 80523, USA; Colorado State University, Department of Agricultural Biology, Fort Collins, CO 80523, USA; University of Maryland, USA

**Keywords:** Gene expression regulation, herbicide resistance, hydroxylation, metabolism, monooxygenase, oxidation

## Abstract

Evolution of metabolic herbicide resistance is a major issue for weed management. Few genes and regulatory mechanisms have been identified, particularly in dicotyledonous weed species. We identified putative causal genes and regulatory mechanism for tembotrione resistance in Palmer amaranth (*Amaranthus palmeri*). Cytochrome P450 candidate genes were identified through RNA-seq analysis. We validated their functions using heterologous expression in *Saccharomyces cerevisae*. Promoters of the candidate P450 genes were analysed. We performed QTL mapping to identify genomic regions associated with resistance. CYP72A1182 metabolized tembotrione in a heterologous system. The *CYP72A1182* gene had increased expression in other *A. palmeri* populations resistant to multiple herbicides, including tembotrione. Resistant plants exhibited polymorphisms in the promoter of *CYP72A1182*. We identified quantitative trait loci linked to herbicide resistance, including one on chromosome 4 approximately 3 Mb away from *CYP72A1182*. *CYP72A1182* is likely involved in tembotrione resistance in *A. palmeri*. Increased expression of this gene could be due to *cis*-regulation in the promoter, as well as *trans*-regulation from transcription factors, and further studies are in progress to test this hypothesis. The elucidation of regulatory genes is crucial for developing innovative weed management approaches and target-based novel herbicide molecules.

## Teaser

The *CYP72A1182* gene has a functional role in metabolic herbicide resistance in *Amaranthus palmeri* and is linked to *cis* -regulatory polymorphisms, advancing understanding of metabolic resistance in dicot plants.

## Introduction

Evolution of herbicide resistance results from the selection pressure imposed by the frequent and consistent use of herbicides over time (Gaines *et al.*, 2020). The emergence and spread of herbicide-resistant weeds poses a major challenge to sustainable agriculture, as they can diminish crop yields and increase the costs associated with weed control (Oerke, 2006; Chauhan, 2020). Herbicide resistance in plants can be selected through different mechanisms, primarily classified as target-site (TSR) and non-target-site (NTSR) resistance (Gaines *et al.*, 2020; Rigon *et al.*, 2020). Between these two groups, NTSR, specifically metabolic resistance, is a critical issue due to the potential interaction of a single metabolic resistance mechanism with herbicides spanning multiple modes of action (Rigon *et al.*, 2020).

Plants possess a diverse array of enzymes involved in the detoxification of xenobiotics. Among these enzymes, cytochrome P450 enzymes (P450s) play a key role in the initial step of the detoxification process (Rigon *et al.*, 2020). P450s associated with herbicide metabolism have been identified from various crops (Siminszky *et al.*, 1999; G. Pan *et al.*, 2006; Kato *et al.*, 2020; Brazier-Hicks *et al.*, 2022; L. Pan *et al*., 2022) and implicated in evolved herbicide resistance in weeds. Notable examples from weeds include *CYP81A12*, *CYP81A21*, *CYP81A15*, and *CYP81A14* from *Echinochloa phyllopogon* (Dimaano *et al.*, 2020); *CYP81A69* and *CYP81A70* from *Cynodon dactylon* (Zheng *et al.*, 2022); *CYP81A10v7* from *Lolium rigidum* (Han *et al.*, 2021); and *CYP77B34* from *Descurainia sophia* (Shen *et al.*, 2022). Nearly all of the identified resistance-conferring genes from weeds to date are from grass species (Rigon *et al.*, 2020).

Palmer amaranth (*Amaranthus palmeri*) is a highly invasive and troublesome weed that poses a significant threat to agricultural crops and ecosystems. Native to the southwestern USA, this annual dicot weed has become a pervasive problem across North America and globally due to its aggressive growth, adaptability, and resistance to herbicides (Ward *et al.*, 2013; Roberts and Florentine, 2022). *Amaranthus palmeri* is dioecious, with separate male and female plants, obligate cross-pollination, and high within- and among-population genetic diversity (Ward *et al.*, 2013; Gaines *et al.*, 2021). Populations have evolved resistance to herbicide spanning nine different modes of action, including synthetic auxins (Foster and Steckel, 2022; Shyam *et al.*, 2022), glufosinate (Priess *et al.*, 2022), and herbicides targeting acetolactate synthase (ALS) (Berger *et al.*, 2016), glyphosate (EPSPS) (Gaines *et al.*, 2010), protoporphyrinogen oxidase (Montgomery *et al.*, 2021), photosystem II (Jhala *et al.*, 2014), 4-hydroxyphenylpyruvate dioxygenase (HPPD) (Küpper *et al.*, 2018), very long chain fatty acid elongase (Hwang *et al.*, 2023), and tubulin biosynthesis (González-Torralva and Norsworthy, 2021).

*Amaranthus palmeri* has evolved herbicide resistance through two main mechanisms, TSR and NTSR. The most commonly observed cases of resistance involve TSR to ALS and EPSPS inhibitors (Gaines *et al.*, 2010; Palmieri *et al.*, 2022). However, there are increasing resistance cases due to metabolic-based mechanisms (Küpper *et al.*, 2018; Shyam *et al.*, 2022), and further analysis is necessary to identify the specific genes involved and their regulation. RNA-seq analysis plays a crucial role in deciphering the genetic basis of herbicide resistance, especially when resistance is a multigenic trait (Gaines *et al.*, 2014; Kohlhase *et al.*, 2019). Additionally, with the availability of complete genomes for weed species (Montgomery *et al.*, 2024), such as *A. palmeri*, conducting quantitative trait locus (QTL) mapping experiments becomes a valuable approach for determining the genetic architecture of resistance (Murphy *et al.*, 2021). By combining comprehensive gene expression data obtained from RNA-seq analysis with QTL mapping, researchers can gain valuable insights into the genetic factors underlying resistance and other important characteristics in weeds.

This research aimed to identify and functionally validate metabolic genes associated with resistance to tembotrione (an HPPD inhibitor) within a particular population of *A. palmeri*. We subsequently sought to determine whether an identified gene, *CYP72A1182*, was also associated with resistance in other suspected tembotrione-resistant *A. palmeri* populations. The research also sequenced the promoter and ran a QTL analysis to investigate potential regulation of *CYP72A1182*.

## Materials and methods

### Plant material for QTL mapping and RNA-seq analysis

Resistant (Nebraska resistant, NER) and susceptible (Nebraska susceptible, NES) *A. palmeri* populations were collected from fields in Shickley, Nebraska in 2011. NER is resistant to atrazine and the HPPD inhibitors tembotrione, mesotrione, and topramezone (Jhala *et al.*, 2014; Küpper *et al.*, 2018). The enhanced metabolism of tembotrione to hydroxy-tembotrione was identified as the mechanism of herbicide resistance in the NER population (Küpper *et al.*, 2018). To minimize the impact of genetic differences unrelated to metabolic herbicide resistance, QTL mapping and RNA-seq was performed on pseudo-F_2_ plants generated from controlled pairings of NER and NES parents.

For the crosses, *Amaranthus palmeri* seeds were sown on 0.7% agar medium (Sigma-Aldrich), placed in a refrigerator at 4 °C for 7 d and then germinated on a germination bench at room temperature with a 16/8h day/night cycle. Germinated seedlings were transplanted into commercial potting soil (Professional Growing Mix, Sun Gro Horticulture) in 5×5 cm inserts and maintained in the greenhouse at 24±2 °C temperature and 15/9 h day/night photoperiods supplemented with metal-halide lamps (400 µmol m^−2^ s^−1^). Plants were watered daily. A pseudo-F_2_ generation was generated by first spraying parental NER individuals at 7–10 cm height with a recommended field rate of 91 a.i. ha^−1^ tembotrione (Laudis, Bayer CropScience) and 1% v/v methylated seed oil (MSO). Herbicide applications were made using an overhead track sprayer (DeVries Manufacturing) equipped with a flat-fan nozzle tip (TeeJet 8002EVS, Spraying System) calibrated to deliver 187 liters ha^−1^ of spray solution at 172 kPa. Surviving NER were transplanted into 22.5 cm diameter pots and individually crossed with another NES individual using pollination bags. Five crosses with NER male × NES female and five crosses with NER female × NES male were performed and grown to seed. The resulting F_1_ generations were grown out and sprayed again under the same conditions described above. Two F_1_ individuals from each parental cross that survived the application were crossed with each other. Seeds from the resulting pseudo-F_2_ generation were subsequently used for the QTL mapping and RNA-seq experiment. Schemes of the crosses are available in Supplementary Fig. S1.

### RNA-sequencing analysis

For the RNA-seq experiment, plants were grown from the pseudo-F_2_ generations generated from two parental NER male × NES female crosses (cross A and B, respectively). Seeds were sown on 0.7% agar medium (Sigma-Aldrich), placed in a refrigerator at 4 °C for 7 d, and then germinated on a germination bench at room temperature with 16/8 h of day/night cycle. About 150 seedlings from each pseudo-F_2_ cross were transplanted into 4×4 cm inserts each and maintained in a growth chamber at 25/22 °C day/night temperatures, 70% relative humidity, and 16 h photoperiod with 700 μmol m^−2^ s^−1^ provided by incandescent and fluorescent bulbs. At 4–5 cm height and four- to five-leaf stage, the first fully expanded leaf from the apical meristem of each plant was cut and immediately frozen at −80 °C for time point 0. The plants were then sprayed at 77 g a.i. ha^−1^ (85% of the field rate) with 1% v/v MSO as described above to ensure resistant plants would be able to survive despite removing leaves for testing. Six hours after herbicide treatment (HAT) the second expanded leaf and 12 HAT the third expanded leaf were cut and immediately frozen at −80 °C. Visual damage and survival data were recorded after treatment for 21 d of phenotype resistant and susceptible individuals. From each pseudo-F_2_ cross only the six visually most resistant and the six visually most susceptible individuals were used for RNA-seq.

Total RNA was extracted from frozen ground tissue using the Direct-zol RNA MiniPrep Plus (Zymo Research) which includes DNAse treatment. Yield and purity were measured with a NanoDrop 2000 spectrophotometer (Thermo Fisher Scientific) and RNA integrity was measured on an Agilent 2200 Bio TapeStation system (Agilent Technologies) using Agilent High Sensitivity RNA ScreenTape. RNA-seq library preparation was performed with the TruSeq stranded mRNA library prep kit (Illumina) preparing for 150 nucleotide paired-end sequencing. The 108 libraries were run on an Illumina HiSeq 4000 platform on a total of 16 lanes (two flow cells) with seven libraries per lane, yielding 5.2 billion paired-end reads. Individual library yields were 48.2 million on average and ranged from 35.0 million to 71.5 million paired-end reads. On average over 93% of sequenced nucleotides met a quality score of 30 (Q30 Phred score).

Raw reads were pre-processed by removal of library adapter sequences, removal of low quality reads, and assessing the quality control using fastp (Chen *et al.*, 2018). Read alignment of the 108 libraries to the available male genome of *A. palmeri* (v1.1, id55760; Montgomery *et al.*, 2020) was performed using Hisat2.2.1 (Kim *et al.*, 2019). Most of the reads (>77%) aligned concordantly once, while >7% aligned concordantly more than one time and about 14% of reads aligned non-concordantly. Mapped reads were assigned to gene features using featureCounts (Liao *et al.*, 2014).

### P450 gene sequences and phylogenetic tree

Consensus sequences of the candidate P450 genes were extracted from the transcriptome and aligned to the reference *A. palmeri* genome (Montgomery *et al.*, 2020) to analyse single nucleotide polymorphisms (SNPs). PCR was performed to amplify the coding sequences in four each of sensitive (S) and resistant (R) plants from the pseudo-F_2_ population that were used for the RNA-seq experiment. cDNA synthesis was performed using ProtoScript II First Strand cDNA synthesis kit (New England Biolabs) with 1 µg of RNA. Protein sequences from cytochrome P450 known to metabolize herbicides in different plant species were obtained from NCBI. Multiple protein alignment was performed using ClustalO and used for tree construction with the neighbor-joining method using Geneious Prime 2023.0.1.

### Quantitative reverse transcription–PCR validation

NES and NER populations were grown and tembotrione herbicide was applied at 91 g a.i. ha^−1^ as previously described for the RNA-seq experiment. Four plants of each population were used for gene expression analysis validation. The youngest leaf tissue was collected at 0, 3, 6, and 12 HAT in 2 ml Eppendorf tubes and placed in liquid nitrogen. The tubes were kept at −80 °C for further analysis. Tissue was ground with 3 mm stainless steel beads in a TissueLyser (Qiagen) with intensity of 30 for 1 min. RNA was isolated using Direct-zol RNA Miniprep from Zymo Research. cDNA synthesis was performed using ProtoScript II First Strand cDNA synthesis kit using 1 µg of RNA and purified with DNase I.

Relative gene expression was analysed on a T100 Thermal Cycler (Bio-Rad Laboratories), using SsoAdvanced universal SYBR® Green supermix. Reaction mixtures consisted of 10 µl of SsoAdvanced universal SYBR® Green supermix (2×) and 2.5 µl of forward and reverse primers at 10 µM, and 5 µl of cDNA (1:20 dilution). Thermocycler conditions consisted of an initial step of 30 s at 95 °C followed by 35 cycles of 5 s at 94 °C and 30 s at 60 °C. Melt curve analysis was added using 65 °C with 0.5 °C increments of 5 s per step.

The reference genes used were *18S rRNA* and *Actin7*. These two genes had the best gene stability as assessed by the NormFinder algorithm (Andersen *et al.*, 2004; Supplementary Table S1). Primers for the reference genes were designed based on conserved regions after alignment of *18S* (*FJ669720.1*), *actin* (HQ656028.1), and *TUB* (XM_010693569.3) from *Beta vulgaris* with the reference genome from *A. palmeri* (CoGe—v1.1, id55760). The candidate genes tested based on RNA-seq results were *CYP72A1182*, *CYP72A1027*, *CYP72A1015*, and *CYP81CJ2*. Another *CYP72A-like* gene, hereafter named as *CYP72A*_*4286* was used as a cytochrome P450 not associated with herbicide resistance based on RNA-seq data. Primers for candidate genes were designed on regions based on consensus sequences from the transcriptome and Sanger sequencing of the genes and are listed in Supplementary Table S2.

### Heterologous expression of CYPs

The genes of interest were transformed and expressed in yeast for further investigation of their role in tembotrione metabolism. Coding sequences of the candidate genes were synthesized, optimized for yeast transformation, and inserted in the pUC-WG/ampv vector by GENEWIZ (Azenta Life Sciences). The gene sequences used were *CYP72A1182* allele 1 (GenBank accession number: OR596705) and 2 (OR596706), *CYP72A1027* (OR596707), *CYP72A1015* allele 1 (OR596708) and 2 (OR596709), and *CYP81CJ2* allele 1 (OR596710) and 2 (OR596711). The gene *CYP81A9* from maize was previously identified as the major locus conferring resistance in maize to the herbicides nicosulfuron, mesotrione, and tembotrione through enhanced herbicide metabolism (Williams and Pataky, 2008). This P450 gene, also known as *Nsf1* for *Nicosulfuron resistance 1*, was included in heterologous expression experiments as a positive control expected to hydroxylate the herbicide tembotrione. Restriction sites for *Bam*HI and *Eco*RI were added to the 5′ and 3′ ends, respectively. Kozak sequence (AAAAAATCT) was added at the 5′ end as a protein translation initiation site. Restriction enzyme reactions were performed to cut the optimized sequence from the vector using 1 μl *Eco*RI, 1 μl *Bam*HI, 500 ng of the vector, with incubation at 37 °C for 2 h. Reaction products were run on 1% agarose gel electrophoresis and the gene band was isolated and purified using a DNA Gel Purification Kit from New England Biolabs.

The pYES2 yeast expression vector was used for recombinant expression. It contains the *URA3* gene for selection in yeast and 2µ origin for high-copy maintenance and GAL1 promoter to express protein (Thermo Fisher Scientific). The ligation reaction was performed using LigaFast Rapid DNA Ligation System from Promega. The reaction consisted of 50 ng digested pYES2 plasmid vector, 5 μl 2× Rapid Ligation Buffer, 1 μl T4 DNA ligase (3 Weiss unit μl^−1^), 50 ng of gene, and purified water up to 10 µl. The reaction was incubated overnight at 4 °C. The product from the reaction was used to transform *Escherichia coli* using the One Shot TOP 10 kit (Thermo Fisher Scientific). The transformed cells were plated with ampicillin (100 μg ml^−1^) and incubated at 37 °C overnight. Single colonies were selected, and gene insertion was confirmed by colony PCR using high-fidelity PrimeSTAR HS DNA Polymerase (Takara). The gene sequence was confirmed by Sanger sequencing. Plasmids were isolated using ZymoPure II Plasmid Miniprep Kit (Zymo Research).

### Yeast transformation and tembotrione incubation

The strains WAT11 and WAT21 of *Saccharomyces cerevisiae* expressing Arabidopsis genes for cytochrome P450 reductase 1 and 2 (Urban *et al.*, 1997), respectively, were used as a heterologous system to test the hypothesis that the candidate P450 genes could metabolize tembotrione. Yeast cells were grown in glucose SC (−Ura) agar plates. Single cells were inoculated in YPD(A) medium and transformed using a modified lithium acetate procedure (Gietz *et al.*, 1992). Transformed yeast cells were selected by glucose SC (−Ura) agar plates. Yeast colony PCR to confirm the gene insertion was performed using high-fidelity PrimerStar HS DNA polymerase by heating the master mix at 94 °C for 4 min before the PCR protocol.

Single colonies containing empty pYES2 or the candidate CYP genes were incubated in 15 ml 2% raffinose medium and allowed to grow for 2–3 d at 30 °C. Cell density was measured spectrophotometrically until the OD_600_ in 20 ml of induction medium was 5. The volume was removed and pelleted at 1500 *g* for 15 min at 4 °C. The cells were resuspended with 1 ml of induction medium containing 2% galactose and inoculated into 20 ml of the same medium. Tembotrione at 1500 μM diluted in ethanol was applied right after the cells were incubated in the induction medium. The yeast cells were incubated at 30 °C at 200 rpm. Twenty-four hours after herbicide application, 5 ml of the medium was collected in 15 ml falcon tubes and cells were pelleted at 1500 *g* for 5 min. The supernatant was collected, and cleaned by adding 5 ml of 5% acetic acid acetonitrile + 1 package of QuEChERS (1 g NaCl and 4 g MgSO_4_) (Phenomenex, Torrance, CA90501), vortexed for 30 s, and centrifuged for 15 min at 2000 *g*. The supernatant was collected, filtered through 13 mm by 0.2 µm nylon (Econofltr Nyln, Agilent Technologies), and injected in the liquid chromatography–tandem mass spectrometry (LC-MS/MS) instrument.

### Liquid chromatography–tandem mass spectrometry protocol

The LC-MS/MS system consisted of a Nexera X2 UPLC with 2 LC-30AD pumps, a SIL-30AC MP autosampler, a DGU-20A5 Prominence degasser, a CTO-30A column oven, and SPD-M30A diode array detector coupled to an 8040 quadrupole mass spectrometer. For tembotrione, the MS was in negative mode with multiple reaction monitoring (MRM) optimized for 439.1>226.05 and set for 100 ms dwell time with a Q1 pre-bias of 11.0 V, a collision energy of 11.0 V and a Q3 pre-bias of 14.0 V. For hydroxy-tembotrione, the MS was in negative mode with MRM optimized for 455.1>419.05 and set for 100 ms dwell time with a Q1 pre-bias of 11.0 V, a collision energy of 11.0 V and a Q3 pre-bias of 14.0 V. The samples were chromatographed on a 100×4.6 mm Phenomenex Kinetex 2.6 μm biphenyl column maintained at 40 °C. Solvent A consisted of water with 0.1% formic acid and solvent B was acetonitrile with 0.1% formic acid. The solvent program started at 80% B, increased to 100% B in 3.5 min, and was maintained at 100% for 2 min. The solvent was returned to 80% B and maintained there for 3 min before the next injection. The flow rate was set at 0.4 ml min^−1^ and samples were analysed as 1 μl injection volumes.

### *CYP72A1182* and *CYP81CJ2* promoter amplification

Pseudo-F_2_ plants were grown in the greenhouse, and the youngest leaf tissue was collected when the plants reached the four to five true leaf stage. Tembotrione was applied at 77 g ha^−1^ rate. Five each of R and S plants from a pseudo-F_2_ population were chosen for promoter amplification. DNA extraction was performed using the hexadecyltrimethylammonium bromide (CTAB) method (Doyle and Doyle, 1990). DNA quantification was carried out using a Nanodrop 2000c instrument (Thermo Fisher Scientific). Primers were designed by Primer3Plus (https://www.bioinformatics.nl/) using as reference the available draft genome of *A. palmeri* (Montgomery *et al.*, 2020). The primer sequences used are listed in Supplementary Table S2. Reverse primers were designed on the conserved sequence of the first exon for each gene. PCR was performed using PrimeSTAR HS DNA Polymerase kit consisting of 10 µl 5× PrimeSTAR buffer (Mg^2+^ Plus), 4 µl dNTP mix (2.5 mM each), 1.5 µl of each primer (forward and reverse), 0.5 µl PrimeSTAR HS DNA Polymerase, 1 µl of DNA (50 ng), and sterile water up to 50 µl. PCR cycling conditions were initial denaturation at 98 °C for 30 s, followed by 40 cycles of denaturation at 98 °C for 10 s, annealing at 60 °C for 15 s, and extension at 72 °C for 2.5 min. PCR product was run in 1% agarose gel electrophoresis for 30 min and amplicons were sent for sequencing using the long-read sequencing technology Oxford Nanopore Technology (ONT) by SNPsaurus LLC (https://www.plasmidsaurus.com). Sequence results were confirmed by blasting the reads to the *A. palmeri* genome and submitted to analysis for common or different motifs using the Multiple Expectation maximizations for Motif Elicitation (MEME-suite) tool (Bailey and Elkan, 1994). Nucleotide motifs were scanned for biological roles using the Gene Ontology for Motifs (GOMo) tool (Buske *et al.*, 2010) to determine if any motif was significantly associated with genes linked to one or more gene ontology (GO) using the Arabidopsis database.

### Involvement of *CYP72A1182* in different tembotrione-resistant *A. palmeri* populations

Seeds from *A. palmeri* populations were collected from agricultural fields in 2019 in the USA with suspected herbicide resistance to mesotrione and tembotrione. Whole-plant dose–response, [^14^C]tembotrione metabolism, P450 gene expression, and gene copy number were evaluated to test the hypothesis that the same candidate P450 gene is involved in the resistance mechanism in these additional populations. Initial screening was performed in the greenhouse using one and two times the label rate of mesotrione and tembotrione to confirm herbicide resistance. Ten suspected resistant populations (WR2019-274, WR2019-141, WR2019-140, WR2019-137, WR2019-199, WR2019-200, WR2019-044, WR2019-144, WR2019-198, WR2019-273), two known tembotrione-resistant (WR2013-034 and NER), and one sensitive control (IHX_3361) population were used for the following experiments.

#### Whole-plant dose–response

Seeds from 13 *A. palmeri* populations were sown in plastic trays filled with soil and kept in the greenhouse at 28 °C and photoperiod of 16 h light. After 1 week, two seedlings were transplanted into single fiber pots with dimensions of 11×7×11.5 cm comprising one replicate. The dose–response experiment consisted of 11 doses, which were 0, 1/128, 1/64, 1/32, 1/16, 1/8, 1/4, 1/2, 1, 2 and 4× the label rate (91 g a.i. ha^−1^). Every dose had six replicates, with a total of 12 plants for each dose. The herbicide tembotrione (Laudis, 419 g a.i. l^−1^, Bayer, Leverkusen, Germany) was applied together with 2200 g a.i. ha^−1^ of the wetting agent Mero (Bayer) and 170 g a.i. ha^−1^ ammonium sulfate using a stationary research sprayer (Höchst AG, Frankfurt-Höchst, Germany) calibrated to deliver a spray volume of 300 liters ha ^−1^. Survival and fresh shoot weight were recorded 28 d after application.

#### [^14^C]Tembotrione metabolism

Metabolism of tembotrione was measured in 13 populations over time in six individuals per population and treatment. Tembotrione was applied on the two youngest expanded leaves of individuals at the four-leaf stage with a total of ten 1 μl droplets (5 μl per leaf) of [^14^C]tembotrione (Bayer) in a 0.3% v/v Mero solution (Bayer) with 3.3 kBq or 200 000 dpm μl^−1^, corresponding to 0.762 μg μl^−1^ of tembotrione. Treated plants were kept in a growth chamber at 28 °C under continuous light conditions with a light intensity of 500 μmol m^−2^ s^−1^ and 70% humidity. Plant shoots were harvested at 6, 12, 24, and 48 HAT. The harvested tissue was washed in 80% acetone three times to remove any non-absorbed [^14^C]tembotrione, and then disrupted in 500 μl of methanol with 5 mm stainless steel beads at 30 Hz for 10 min. The homogenate was centrifuged at 6000 *g* for 10 min. The residue was re-extracted with 600 μl of methanol followed by a final extraction with 600 μl of 90% acetonitrile. All solvents used were high-performance liquid chromatography (HPLC) grade (Sigma-Aldrich, Steinheim, Germany; ≥99.9% HPLC grade). The pooled supernatant was evaporated under continuous air flow at 55 °C, re-suspended in 200 μl of 90% acetonitrile using a shaker and ultrasonic bath, and then filtered through a 0.45 μm low-binding hydrophilic polytetrafluoroethylene mesh for 10 min at 2200 *g* in the centrifuge. The recovered radioactivity in the filtrate was 92% of the total applied, on average. A non-treated control sample, spiked with [^14^C]tembotrione just prior to extraction, was also included. Separation and HPLC identification of the parent tembotrione herbicide and its metabolites were performed on a reverse-phase HPLC system (LC Net II/ADC with PU-980 pump unit, LC-980-02 gradient unit and CO-2060 Plus column thermostat; Jasco, Oklahoma City, OK, USA). Chromatographic separation was achieved with a 150×2.0 or 3.0 mm internal diameter Luna C18 (2) column with a particle size of 3 μm (Phenonemex, Aschaffenburg, Germany) at a flow rate of 0.5 ml min^−1^. The mobile phases consisted of 0.05% phosphoric acid (A) and acetonitrile: 0.05% formic acid (B) and were run at a 60 min linear gradient from 0 to 60% solvent B, followed by a 1 min linear gradient from 60% to 90% solvent B, plateauing for 4 min. The column was then flushed with 100% solvent A for 7 min.

#### P450 gene expression

To ascertain the involvement of *CYP72A1182* in the newly identified resistant populations, a gene expression experiment was conducted. The aim was to assess the expression levels of *CYP72A1182* and determine its potential role in conferring resistance within the populations. Plant growth and herbicide application at 91 g a.i. ha^−1^ were performed as described previously. The NES population (Küpper *et al.*, 2018) was used as a second negative control in this experiment. Four plants of each population were used for gene expression. Tissue of the first and second youngest leaves were harvested before and 6 HAT, respectively. Tissue was collected into 2 ml Eppendorf tubes and placed in liquid nitrogen. The tubes were kept at −80 °C for further analysis. Tissue was ground with 3 mm stainless steel beads in a TissueLyser (Qiagen) with intensity of 30 for 1 min. RNA was isolated using Direct-zol RNA Miniprep (Zymo Research) and purified with DNase I. cDNA synthesis was performed using a ProtoScript II First Strand cDNA synthesis kit using 1 μg of RNA. Gene expression analysis and conditions were the same as defined in the section of gene validation. The reference gene used in the experiment was *18S rRNA*, and the candidate genes tested were *CYP72A1182* and *CYP81CJ2*.

#### Gene copy number

The six *A. palmeri* populations most resistant to tembotrione described above, along with NES, NER, and another sensitive population, IHX_3361, were used for *CYP72A1182* gene copy number analysis. Plant growth, tissue collection, and DNA extraction were performed as described. Genomic DNA at 50 ng/μl was used for relative gene copy quantification using a modified method $2^{-\Delta \Delta C_{t}}$ (Livak and Schmittgen, 2001). The *ALS* gene was used as a single-copy control gene. The primers for *ALS* were used from previous research studying *EPSPS* copy number in *A. palmeri* (Gaines *et al.*, 2010). Relative quantification of *CYP72A1027* was calculated with a modified Δ*C*_t_ method (*C*_t,ALS_−*C*_t,CYP_). qPCR conditions were the same as described in the section for gene expression validation. Each population had eight biological samples run in two technical replicates.

### Mapping of tembotrione resistance in NER *A. palmeri* population

#### Library preparation and QTL identification

A dose–response experiment was conducted to define a delimiting rate to best differentiate the S×S (NES) and R×R (NER) populations. Seed of NES, NER, and two F_1_ populations NER male × NES female (crosses A and B) were sown on soil and germinated in the greenhouse at 28 °C and 16 h light photoperiod then transplanted to 4×4 cm inserts after 7 d. The dose–response experiment consisted of 11 doses, which were 0, 1/128, 1/64, 1/32, 1/16, 1/8, 1/4, 1/2, 1, 2 and 4× the label rate (91 g a.i. ha^−1^). Each dose was applied on two plants with eight replicates. The herbicide tembotrione (Laudis, 419 g a.i. l^−1^, Bayer) was applied together with 1% v/v MSO using an automated spray chamber (Greenhouse Spray Chamber, model Generation IV) using a TJ8002E nozzle, calibrated to deliver 200 liters ha^−1^ at a pressure of 280 kPa and speed of 1.2 m s^−1^. Survival and fresh shoot mass were recorded 28 d after application and fitted to a three-parameter log-logistic model using the drc package in R (Ritz *et al.*, 2015).

Segregation in the pseudo-F_2_ population was performed in response to a delimiting rate of 77 g ha^−1^ with 606 plants in cross A and 1277 plants in cross B. At 28 d after tembotrione application, plants were rated and shoot fresh mass was measured. For QTL analysis, the 71 and 110 most susceptible plants and 49 and 91 most resistant plants, from cross A and B, respectively, were selected based on survival and shoot fresh mass. Parental NES and NER were grown and submitted to the same herbicide screen and 20 of each population were selected for QTL mapping.

Plant tissue was collected before herbicide application for DNA extraction from single leaves following a modified CTAB method. DNA samples were assessed for quality using Qubit. Double-digest restriction site-associated DNA sequencing (ddRADseq) libraries were generated with ApeKI and sequenced using NovaSeq S4 with 150 bp paired-end reads (Illumina). The average yield was around 7.4 M reads per library and the mean quality scores were over Q30 for all libraries. The sequencing was performed at the University of Minnesota Genomics Center.

The ddRAD-seq libraries were trimmed using trimmomatic v0.36 (Bolger *et al.*, 2014), aligned to the reference male genome of *A. palmeri* (scaffold file v1.1, id55760; Montgomery *et al.*, 2020) with Burrows–Wheeler aligner (Li and Durbin, 2009), and variants called using GATK 4.2.0 (Poplin *et al.*, 2018, Preprint). Samples were computationally binned by shoot fresh mass. The variant sites were separated by SNPs and indels, and hard filter was performed as follows: for SNPs, QD<2.0, QUAL<30.0, SOR>4.0, FS>20.0, MQ<50.0; and for insertion/indels, QD<2.0, QUAL<30.0, FS>200.0. The variant sites were filtered based on depth (at least five reads) and only variants that were homozygous were selected. The R/qtl2 package was used to find QTLs (Broman *et al.*, 2019). The analysis was performed separately for two distinct crosses (A and B) and combined A+B. QTL intervals were calculated using Bayes credible intervals with the function bayes_int(). To determine the threshold for identifying potential QTLs, 1000 permutation tests were conducted at a confidence level of 95%. The critical *F*-value obtained from this analysis was used as the criterion to declare a putative QTL. The functional analysis of the genes present in QTLs was conducted using DAVID Bioinformatics Resources v6.8 (Sherman *et al.*, 2022).

### Statistical analysis

The alignment of the RNA-seq experiment was used to analyse differentially expressed genes (DEGs) using the DEseq2 (v1.20.0) (Love *et al.*, 2014) package in R (v4.0.4) (R Core Team, 2023). Populations S and R were considered as factor of conditions. Samples from cross A and cross B were pooled together for the analysis. Contrast analyses performed were R versus S before treatment (0 HAT), and 6 and 12 HAT, and 6 HAT versus 0 HAT and 12 HAT versus 0 HAT for each population. The data were submitted for shrinkage log2 fold-change. Counts were normalized by creating a ‘virtual reference sample’ using the geometric mean of counts over all samples for each gene (Anders and Huber, 2010). Principal component analysis (PCA), volcano and MA plots were performed on the normalized gene expression data. Genes with less than 10 reads were excluded from analysis. A *P*-adjusted value <0.05 cutoff and log2 fold-change of 1 was used to identify DEGs.

The validation of candidate P450 genes in NER and NES was performed in four biological samples and two technical replications. Analysis of the *CYP72A1182* and *CYP81CJ2* role in the field-collected *A. palmeri* populations from 2019 was performed using four biological samples and two technical replications. The mean *C*_t_ values and the standard deviation were calculated by treatment. A melt curve analysis confirmed the presence of a single amplified product for each reaction, based on presence of a single melting temperature consistent across samples for each gene. Relative transcript abundance was calculated using $2^{-\Delta \Delta C_{t}}$ method (Livak and Schmittgen, 2001). The reference population used for the P450 validation analysis was NES untreated. To analyse the role of P450 genes in the field-collected populations, the sensitive control (IHX_3361) before herbicide application was used as control. Fisher’s LSD test (*P*<0.05) was used to compare the relative expression between treatments for each experiment.

For the whole-plant dose responses, the statistical software R v.3.5.3 (Ritz *et al.*, 2015) was used for data analysis. Data were adjusted using the three-parameter log-logistic model with the function modelFit () from the drc package (Ritz *et al.*, 2015). The herbicide doses that caused a 50% reduction in each variable were estimated using the model: *y*=*d*/1+exp[*b*(log*x*−log*e*)], where *d* is the upper limit, *b* is the slope, *x* is the dose, and *e* is the dose that causes 50% reduction in *y*. The statistical difference between the resistant biotype and the sensitive biotype for ED_50_ (survival) or GR_50_ (fresh shoot weight) was calculated using the function EDcomp (). The resistance index was calculated using the ratio of GR_50_ values of each biotype with the sensitive biotype. Graphs were generated using GraphPad Prism version 8.2.1 (GraphPad Software, San Diego, CA, USA).

For the [^14^C]tembotrione metabolism analysis, the identification of the primary metabolites—M1, M2, M3, M4, and M5—was based on previous findings (Küpper *et al.*, 2018) and retention time. M3 and M4 represent hydroxylated forms of tembotrione that differ in the position of the hydroxyl group on the left aromatic ring and were therefore combined for analysis. Peak areas (expressed as a percentage of recovered radioactivity) were obtained from HPLC and used to quantify and compare metabolite profiles. The data represent the mean of six replicates per biotype. Means for each biotype were statistically compared with the IHX_3361 (sensitive) population using Dunnett’s multiple comparison test.

## Results

### Differentially expressed genes

A total of 20 846 genes were analysed for differential expression out of the initial set of 29 758 genes assigned by featureCounts. A gene-wise dispersion curve was fitted using the DESeq2 model (Supplementary Fig. S2). There was a favorable dispersion pattern, with decreasing dispersion as the mean expression levels increased, indicating a good fit of the DESeq2 model to the analysed data. The PCA plot revealed that PCA1 and PCA2 accounted for 35% and 21% of the variation in the data, respectively (Supplementary Fig. S3). PCA1 separated cross A from cross B, while PCA2 exhibited separation of gene clusters based on HAT within each cross. When clustering all 72 analysed transcriptome samples, two major clusters emerged, with samples from different crosses grouped together, indicating similarities in gene expression response (Supplementary Fig. S4).

**Fig. 1.**
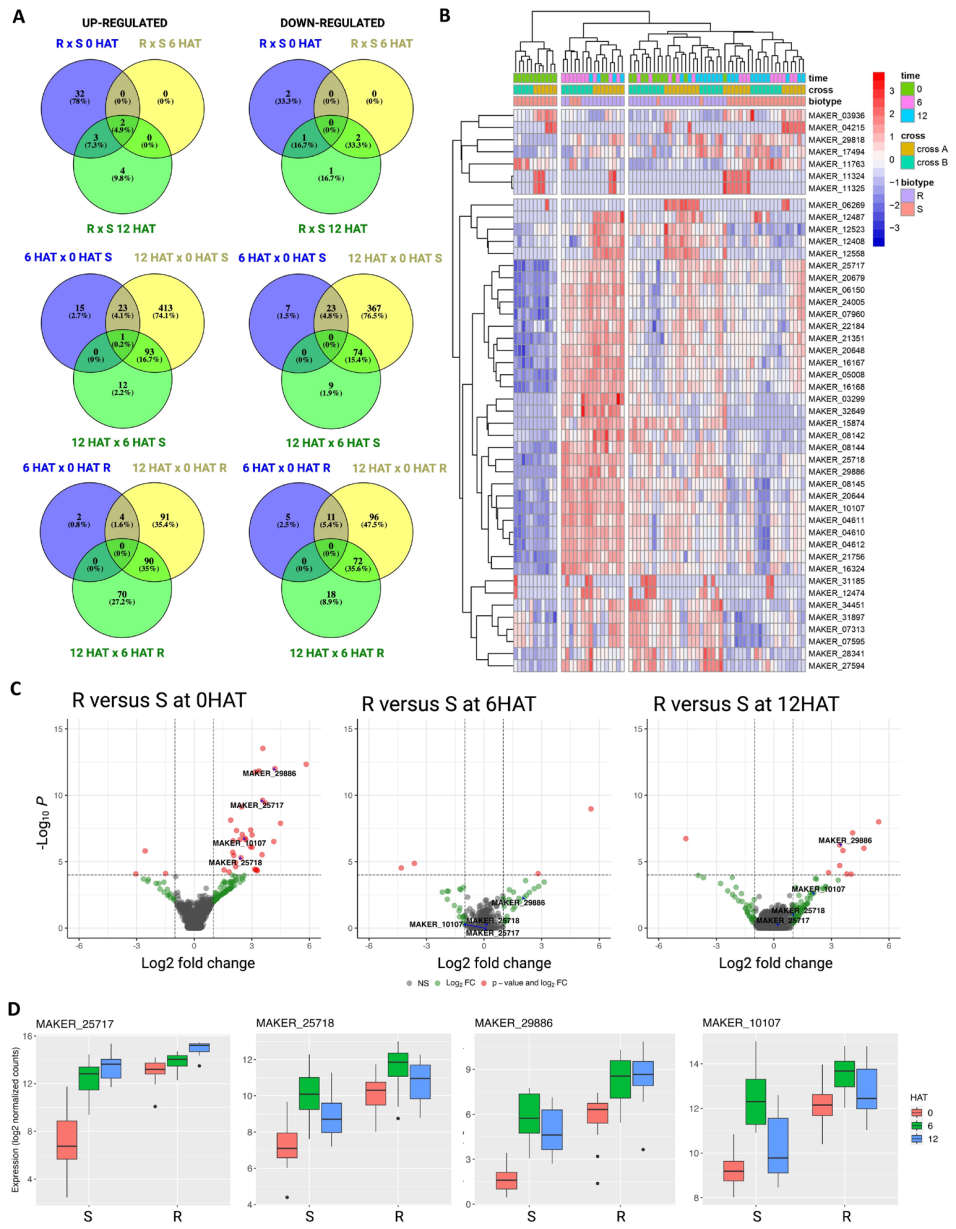
Analysis of differentially expressed genes (DEGs) in pseudo-F_2_*Amaranthus palmeri* susceptible (S) and 4-hydroxyphenylpyruvate dioxygenase (HPPD)-resistant (R). (A) Venn diagram of the up- and down-regulated DEGs indicating unique and common DEGs for 18 different comparisons. (B) Heatmap of top DEGs for the comparisons R vs S at 0, 6, and 12 h after herbicide treatment (HAT). (C) Volcano plots displaying gene expression differences between *Amaranthus palmeri* and treatments of contrast of R versus S before herbicide application (0 HAT), at 6 HAT, and at 12 HAT. DEGs with significant thresholds of *P*<0.0001, adjusted *P*<0.05, log2 fold-change >1 or <−1 are represented by red circles. Dashed lines represent significance thresholds of adjusted *P*<0.05 and log2 fold-change >1 or <−1. (D) Log2 normalized counts of the top four cytochrome P450 genes in response to tembotrione in S and R population, MAKER_25717 (*CYP72A1182*), MAKER_25718 (*CYP72A1027*), MAKER_29886 (*CYP72A1015*), and MAKER_10107 (*CYP81CJ2*). DEGs had a threshold of adjusted *P*-value of 0.05 and log2 fold-change 1.

DEGs were observed in different comparisons. In the contrast comparison of R versus S at constitutive (0 HAT), 6 HAT, and 12 HAT after herbicide application, R had 37, 2, and 9 up-regulated genes and 3, 2, and 4 down-regulated genes, respectively (Fig. 1A). Among these genes, only two genes were commonly up-regulated (Fig. 1A). For the analysis of the genes that were responsive to herbicide application for each phenotype, 39, 530, and 106 genes were up-regulated in S, and 6, 185, and 160 were up-regulated in R for the comparisons of time 6 versus 0 HAT, 12 versus 0 HAT, and 12 versus 6 HAT, respectively (Fig. 1A). The heatmap illustrates the expression of DEGs from these comparisons and indicates a separation in clusters of resistant and susceptible plants (Fig. 1B).

Several genes associated with the detoxification of xenobiotic substances were consistently identified, suggesting an enhanced genetic metabolism in plants with resistance. These genes included four cytochrome P450 (CYP) genes, four glutathione-*S*-transferase genes, seven glycosyltransferase genes, a disease resistance protein, and a detoxification 27 gene (Supplementary Table S3). Additionally, two MADS-box transcription factors of types 23 and 27 were found to be up-regulated, leading to the hypothesis that gene regulation could play an important role in resistance. The resistance mechanism in the resistant population was characterized by increased herbicide metabolism including an initial hydroxylation of the tembotrione molecule (Küpper *et al.*, 2018); therefore, among all DEGs, our focus was on the four cytochrome P450 genes that were consistently up-regulated in the R plants (0 HAT). These genes had the genomic IDs MAKER_29886, MAKER_25717, MAKER_10107, and MAKER_25718, and exhibited up-regulation in the R population with 18.3-, 11.8-, 6.2-, and 5.4-fold changes, respectively, relative to the S population at 0 HAT (Fig. 1B–D; Supplementary Table S3). None of these genes had differential expression at the 6 HAT time point compared with the susceptible plants (Fig. 1C, D), and only one gene (MAKER_29886) exhibited a reactivation at 12 HAT in R population (Fig. 1C, D). This indicates that the susceptible plants responded to the herbicide treatment (Fig. 1C, D), where an increased transcription of these genes was observed at 6 and 12 HAT. However, the resistant plants constitutively expressed these cytochrome P450 genes at a higher level prior to herbicide treatment (Fig. 1C, D).

Constitutively up-regulated P450s, MAKER_25717, MAKER_25718, and MAKER_29886, had significant similarity to *CYP72A219* from *Spinacia oleracea* (NCBI: XM_056843192.1), with identities of 76.2%, 74.3%, and 76.3%, respectively. Similarly, the gene ID MAKER_10107 assigned the name *CYP81CJ2* had high similarity (73.3%) to *CYP81E8* from *Chenopodium quinoa* (NCBI: XP_021724107.1). Hereafter, the genes MAKER_25717, MAKER_25718, MAKER_29886, and MAKER_10107 will be referred to as *CYP72A1182*, *CYP72A1027*, *CYP72A1015*, and *CYP81CJ2*, respectively, based on P450 gene annotation and naming from a recently assembled *A. palmeri* genome (Raiyemo *et al.*, 2025).

### SNPs in cytochrome P450 candidate

Both S and R individuals had two alleles of *CYP72A1182* with a coding sequence of 1544 bp, producing a protein of 517 amino acids. No SNPs were found in the P450 gene sequences that would have consistently caused amino acid differences between S and R individuals. *CYP72A1027* had a coding sequence of 1578 bp and 526 amino acids. Both R and S phenotypes shared the same two alleles for these genes. *CYP72A1015* had a length of 1394 bp and 464 amino acids. Susceptible plants had a single allele while R plants had the same allele and an additional allele with amino acid substitutions of Thr307Ser, Phe388Leu, and Ile407Val. *CYP72A1182* and *CYP72A1027* were localized on chromosome 4 and *CYP72A1015* was localized in chromosome 16. *CYP72A1182* shared identity with *CYP72A1027* and *CYP72A1015* of 76.1% and 55.1%, respectively. Two alleles of *CYP81CJ2* were present in both S and R samples. The gene had a length of 1470 bp and 1479 bp in each allele. The shorter allele had a deletion of 18 bp at position 35, and the longer allele had a deletion of 9 bp at position 37 when aligned to the draft genome. In the available *A. palmeri* genome, this specific region of the gene exhibits a triplication of PPS amino acids, which may be due to incorrect assembly. All cytochrome P450 possess a conserved cluster of proline in the membrane hinge. Similarly, the sequenced cytochrome P450 exhibited this conserved region.

In the phylogenetic tree analysis, *CYP72A* genes from *A. palmeri* clustered with *CYP72A31* from *Oryza sativa*, which can metabolize bispyribac-sodium and bensulfuron-methyl (Saika *et al.*, 2014). *CYP81CJ2* clustered with other P450 of family 81 from grasses but with some significant distances between them (Supplementary Fig. S5). This gene had a low identity when aligned with the other P450s. *ApCYP81CJ2* shared identity of 40.5%, 41.0%, and 39% with *ZmCYP81A9* (Genbank: EU955910.1), *LrCYP81A10v7* (Genbank: MK629521.1), and *EpCYP81A12* (Genbank:AB818460.1), respectively, known to metabolize herbicides in grasses (Brazier *et al.*, 2002; Iwakami *et al.*, 2014; Han *et al.*, 2021; Brazier-Hicks *et al.*, 2022).

### P450 qPCR validation

*CYP72A1182*, *CYP72A1015*, and *CYP72A1015* were more up-regulated in NER plants at 3, 6, and 12 HAT, respectively, in comparison with NES (Supplementary Fig. S6). Among the *CYP72A* genes, *CYP72A1182* had a higher up-regulation at 3 and 6 HAT. Additionally, *CYP81CJ2*, located on chromosome 4, had up-regulation specifically at 6 HAT (Supplementary Fig. S6). These observations deviate from those obtained in the RNA-seq analysis, which indicated that these genes were constitutively more highly expressed in resistant plants (Fig. 1). However, this discrepancy arises due to the utilization of the parental populations NES and NER for gene validation, which exhibit significant individual-level variation in their response to tembotrione. Nevertheless, despite the disparity, the consistency of these findings indicates that the up-regulation of these genes in NER plants differs from that in NES plants.

### *CYP72A1182* metabolizes tembotrione in yeast

**Fig. 2.**
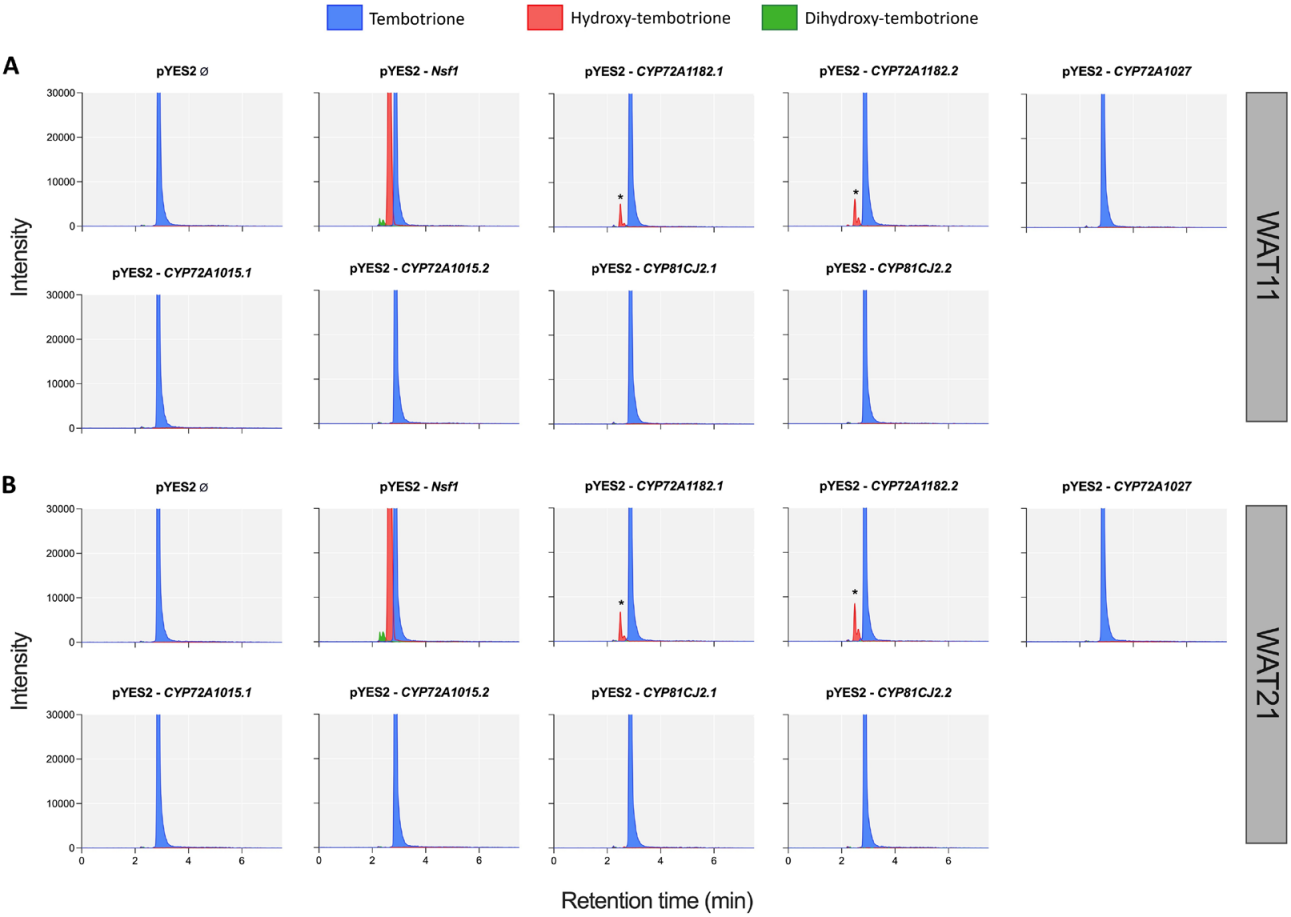
LC-MS/MS chromatogram of metabolites formed in yeast transformed with P450 genes from *A. palmeri* and the *Nsf1* gene from maize as a positive control. Blue peak, parental tembotrione; red peak, hydroxy-tembotrione; green peak, dihydroxy-tembotrione. WAT11 (A) and WAT21 (B) yeast strains carrying Arabidopsis cytochrome P450 reductase 1 and 2, respectively. Asterisk indicates at least 10-fold higher peak than the background signal.

Following a 24 h incubation period with tembotrione, some transformed yeast treatments converted tembotrione into hydroxy-tembotrione. The chromatogram of the empty vector pYES2 revealed solely the presence of the parent tembotrione peak at a retention time of 2.9 min (Fig. 2). The positive control gene *Nsf1* exhibited the expected affinity for tembotrione, producing a hydroxy-tembotrione peak at a retention time of 2.7 min and a dihydroxy-tembotrione peak at 2.5 min (Fig. 2). Among all the candidate P450 genes tested, only *CYP72A1182* resulted in conversion of the parent tembotrione into hydroxy-tembotrione. Both alleles of this gene in the WAT11 and WAT21 strains resulted in metabolism of the herbicide. Conversely, CYP72A1015, CYP72A1015, and CYP81CJ2 did not exhibit any ability to metabolize tembotrione. Based on these results, it can be concluded that of the candidate P450, only CYP72A1182 possesses the capacity to recognize the tembotrione molecule and effectively metabolize it.

### CYP promoter analysis

**Fig. 3.**
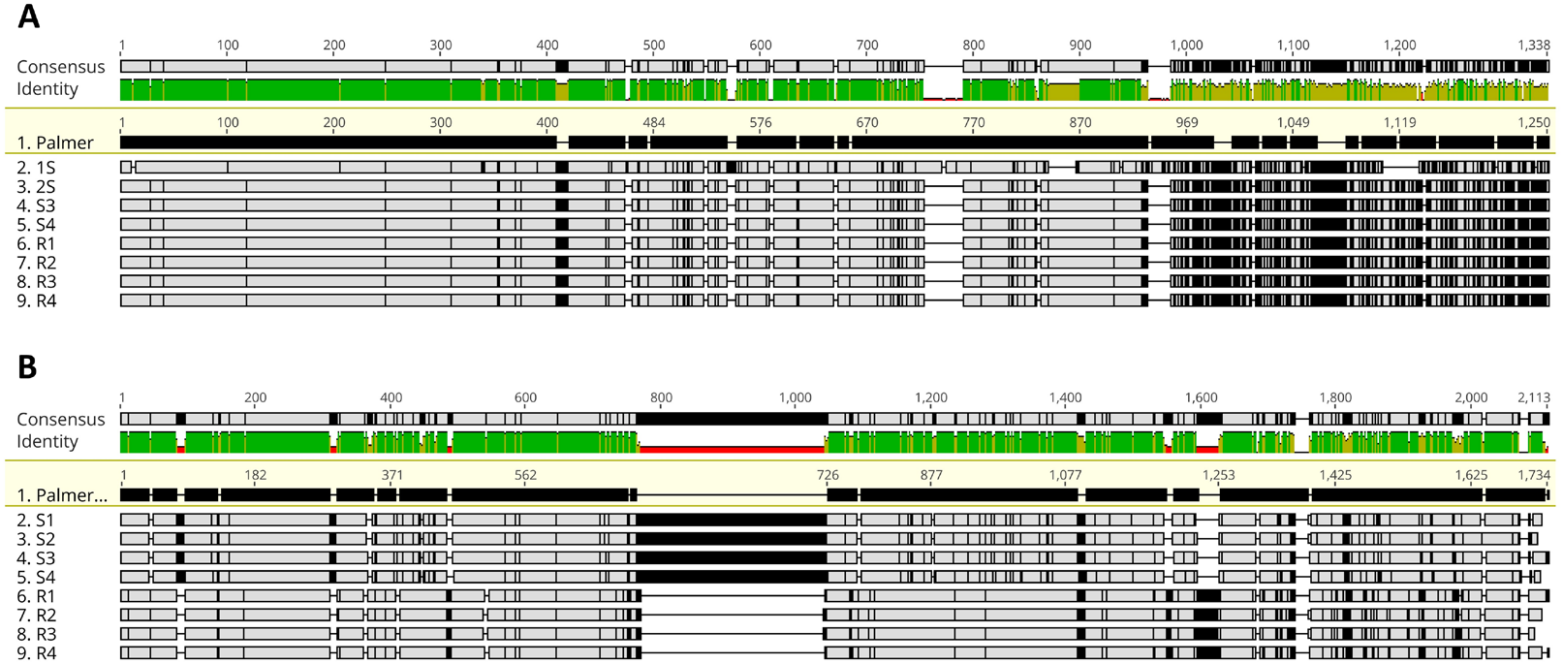
MUSCLE v5 multiple alignment of *CYP81CJ2* (A) and *CYP72A1182* (B) promoter sequences. The promoter sequences were reverse-oriented from the reference genome sequence to enable viewing in + strand orientation. Black space indicates differences from the reference *Amaranthus palmeri* genome (id 55760).

The DNA segment amplified upstream of *CYP81CJ2* was 1250 bp for both the sensitive and resistant plants (Fig. 3). Pairwise sequence alignment between two samples revealed 100% identity between the promoters of S and R (Fig. 3A; Supplementary Fig. S7). For the promoter of the *CYP72A1182* gene on chromosome 4 (MAKER-25717), the primers amplified regions ranging from 1991 to 2004 bp for S plants and from 1747 to 1768 bp for R plants. Pairwise alignment of *CYP72A1182* promoters revealed sequence differences between S and R, with identity of 76.8% (Supplementary Fig. S7). Resistant plants had unique insertions, such as a cytosine at position 45 bp, an 8 bp insertion at position 458 bp, an 8 bp insertion at position 1247 bp, and a 31 bp insertion at position 1291 bp upstream. Sensitive plants, on the other hand, had unique insertions, including a 13 bp insertion at position 82, a 10 bp insertion at position 309 bp, and a 277 bp insertion at position 746 bp upstream of the gene (Fig. 3B; Supplementary Fig. S7).

A total of 15 significant binding-site motifs were identified in the promoter region of *CYP81CJ2*. Interestingly, all motifs were found in both S and R promoters (Supplementary Fig. S8). In the case of the *CYP72A1182* promoter, a total of 25 motifs were discovered. Among these, two motifs were specific to the S promoter, two were specific to the R promoter, and 21 motifs were shared between S and R (Supplementary Figs S8, S9). Notably, the R promoter exhibited replication of four motifs. Some motifs were identified as a binding site for transcription factors such as MYB88 (AT2G02820), suppressor factor AIF1 (AT3G05800), and abiotic stress-responsive transcription factor DREB (AT1G77200) based on GOMo analysis (Supplementary Fig. S9). Furthermore, the motif GTTATTTAGTAACTTWKYGTD, which serves as a binding site for the MYB gene transcription factor, was triplicated in the R promoter (Supplementary Fig. S9).

### Whole-plant dose response, metabolism, and *CYP* expression in different herbicide resistant *A. palmeri*

Based on the whole-plant dose response (Supplementary Fig. S10), the confirmation of *A. palmeri* populations with suspected tembotrione resistance revealed distinct variations, with resistance index ranging from 2.4 to 7.9 (Supplementary Tables S4, S5). Tembotrione metabolism was assessed in the *A. palmeri* populations using ^14^C-lablled herbicide (Supplementary Fig. S11). The method used in the present study was different from the previous study (Küpper *et al.*, 2018), and hence retention times are slightly different. Parental tembotrione had a retention time of 66.9 min and its major metabolites had retention times of 47.3 (M1), 50.1 (M2), 56.3 (M3), 59.0 (M4), and 67.7 min (M5) (Supplementary Figs S12, S13). Hydroxy-tembotrione was represented by two major peaks, M3 and M4. Although both peaks shared the same molecular mass, differences in the position of the hydroxyl group on the left aromatic ring of tembotrione resulted in distinct retention times. NER had higher percentage area peak of hydroxy-tembotrione (M3/M4), the main metabolite associated with herbicide resistance (Küpper *et al.*, 2018), than the sensitive population at 12 HAT (Supplementary Fig. S11). The other resistant populations were similar to NER, except for WR2019-044 and WR2019-273 (Supplementary Fig. S11). The faster hydroxy-tembotrione production in most populations suggests that enhanced metabolism capacity is the main mechanism of HPPD-resistance of these populations. *CYP72A1182* was up-regulated in most resistant populations, either before treatment or 6 HAT (Supplementary Fig. S14). The populations WR2019-034, -137, -140, -199, and -274 had a significantly higher relative *CYP72A1182* expression compared with the sensitive population, ranging from 100- to 658-fold increase. In contrast, the expression of *CYP81CJ2* was not constitutively higher or responsive to tembotrione treatment in *A. palmeri* populations, except in WR2019-140 (Supplementary Fig. S14). The gene copy number of *CYP72A1182* was similar across all resistant populations and to that of sensitive plants (Supplementary Fig. S15). The higher gene expression of *CYP72A1182*, constitutively and after tembotrione application in resistant plants, suggests a crucial role in herbicide resistance in the newly characterized tembotrione-resistant populations.

### Degree of dominance and QTL identification

**Fig. 4.**
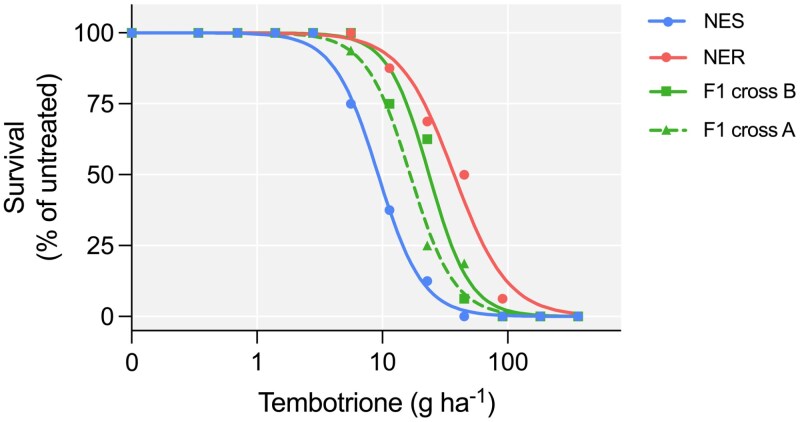
Dose–response curve of *Amaranthus palmeri* herbicide susceptible (NES), resistant (NER), F_1_ cross A, and F_1_ cross B plants in response to tembotrione.

The herbicide dose that caused a 50% reduction in plant survival (ED_50_) of NES and NER was 7.9 g and 37.3 g, respectively (Table 1), lower than previously reported for these populations (Küpper *et al.*, 2018). However, the resistance index between these two populations was similar, 4 compared with 3.3 from the previous study (Küpper *et al.*, 2018). Both F_1_ populations were intermediate between NES and NER in response to tembotrione (Fig. 4). The degree of dominance was calculated and indicated a co-dominant or semi-dominant trait (cross A, 0.42; cross B, 0.67) based on the formula proposed by Bourguet and Raymond (1998). Pseudo-F_2_ populations, resulting from the cross between A and B, exhibited survival rates of 11.4% and 8.8%, respectively, in response to 77 g a.i ha^−1^. These survival rates were similar to the parental homozygous line NER (12.4%), which was cultivated alongside the pseudo-F_2_ plants.

**Table 1.** Logistic equation parameters and resistance index (RI) for survival (% of untreated control) in parental *Amaranthus palmeri* herbicide-susceptible (NES), herbicide-resistant (NER), F_1_ cross A, and F_1_ cross B populations in response to tembotrione

Population	*b*	ED_50_				RI	*P*-value
		Dose (g ha^−1^)	Lower CI (95%)	Upper CI (95%)	SE		
NES	2.4	9.3	7.9	10.6	0.7	—	
NER	2.1	37.3	31.2	43.4	3.1	4	<0.001
F1 cross A	2.4	16.6	14.2	19.1	1.2	1.8	0.2
F1 cross B	2.6	23.6	20.2	27	1.7	2.5	<0.001

b, slope; ED_50_, herbicide dose that causes a 50% reduction in plant survival; CI, confidence interval of parameter ED_50_ (α=0.05); RI, resistance index, which is the ED_50_ ratio between populations with NES.

**Fig. 5.**
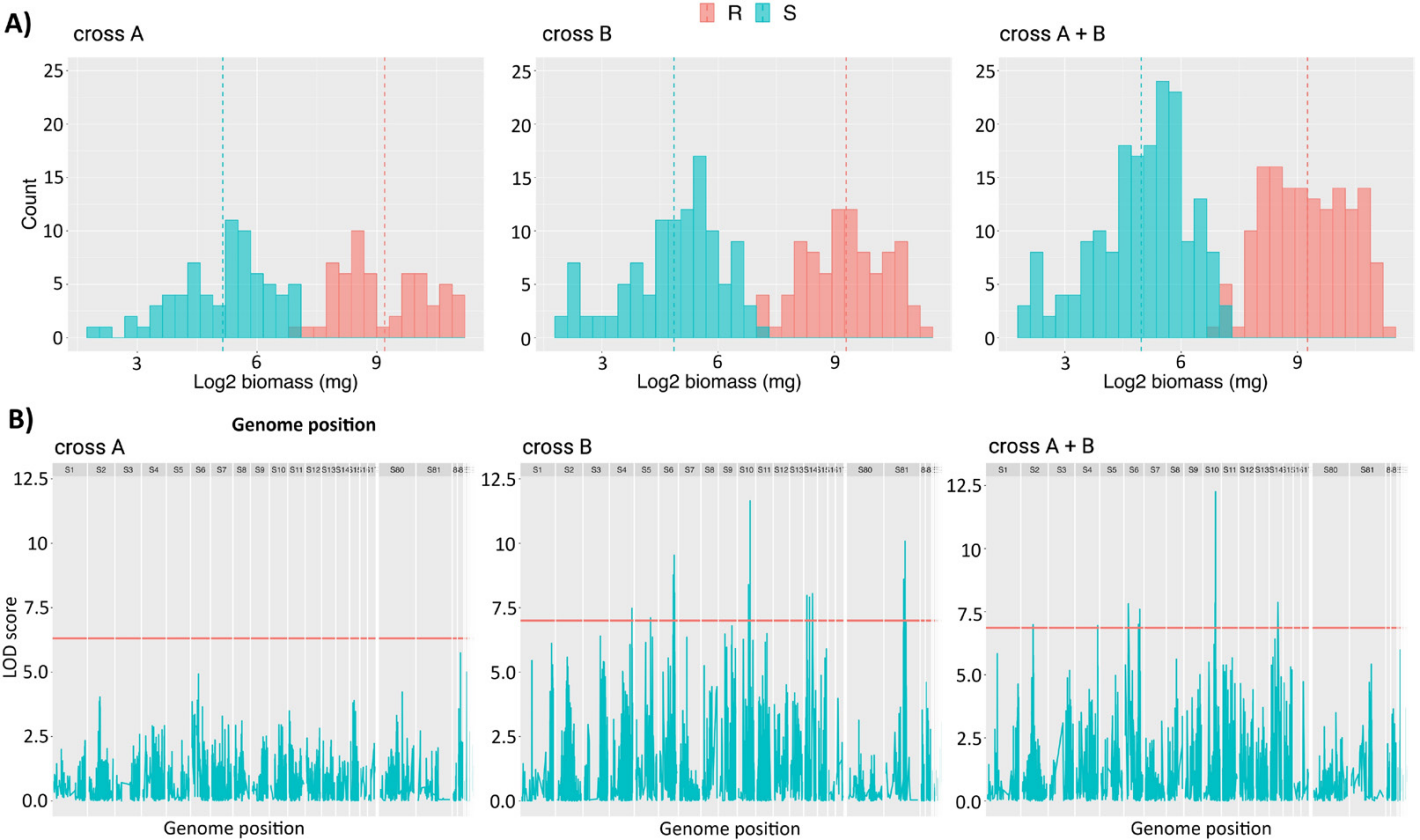
Frequency distribution and genome scan analysis. (A) Distribution of fresh biomass in plants used for QTL mapping of pseudo-F_2_ populations from cross A, B, and combined A+B. Blue and red colors represent sensitive and resistant pseudo-F_2_ plants, respectively. Dashed line indicates the average biomass for parental NES (blue) and parental NER (red). (B) Genome scan of pseudo-F_2_*A. palmeri* populations from cross A, B, and combined A+B. *y*-Axis indicates logarithm of the odds (LOD) score statistical estimate, *x*-axis indicates genome position on scaffolds levels. The plants were treated with 77 g ha^−1^ of tembotrione, and fresh biomass was measured after 28 d.

The frequency distribution of fresh weight after herbicide treatment on pseudo-F_2_ plants had two distinct peaks for each cross, with one peak each indicating phenotypic similarity with each parental population. A normal distribution of R plants had higher biomass (Fig. 5A, red bars) and the same was identified for S samples (Fig. 5A). The variant calling analysis identified over 3.4 million SNP sites in the *A. palmeri* genome. After filtering, a total of 4404 SNP variants were detected throughout the entire *A. palmeri* genome. Genome scanning in cross A did not detect any QTLs surpassing the logarithm of the odds (LOD) threshold of 6.4 after conducting a permutation test. However, when examining pseudo-F_2_ plants from cross B, significant QTLs with high effects were found in multiple scaffolds exceeding the LOD threshold of 6.7. The scaffolds include scaffold 10 with LOD of 11.6, scaffold 81 with LOD of 10.1, scaffold 6 with LOD of 9.5, and scaffold 14 with LOD of 8. Each QTL was accompanied by other significant QTLs nearby (Fig. 5B; Supplementary Table S6). For instance, in scaffold 10, there were three QTL peaks. The biggest peak had a LOD score of 11.6 at position 11 877 263, and two other peaks had LOD scores of 8.4 and 7.9 at positions 10 336 852 and 10 337 656, respectively. The same happened in scaffolds 81, 6, and 14 with nearby peaks. Combining the datasets from cross A and cross B increased the sample size and enhanced the statistical power for the analysis. As a result, the genome scan revealed a more robust QTL effect on scaffold 10, precisely at the same genomic location 11 877 263. The QTL exhibited a higher LOD score of 12.2, indicating a stronger and more significant association with the resistance trait (Supplementary Table S6). Additional QTLs were found on scaffolds 6 and 14, but with lower effects.

The QTL located in scaffold 10 accounted for 23% of the phenotypic variation observed in the data for cross B. Additionally, the QTLs in scaffold 81, 6, and 14 explained 20%, 19%, and 16% of the phenotypic variation, respectively. However, when the data from both cross A and cross B were combined, the overall phenotypic variation explained by the QTLs slightly decreased. This can be attributed to the absence of any QTL in cross A. Specifically, for the combined data, the QTL in scaffold 10 explained 15% of the phenotypic variation, while the QTLs in scaffold 6 and 14 accounted for 10% each (Supplementary Table S6).

To conduct further analysis, functional annotation was performed on the combined dataset due to its larger number of genes and sample size. The QTLs found in scaffolds 10, 6, and 14 were localized on chromosomes 4, 8, and 15 of *A. palmeri*, respectively, and encompassed 82, 196, and 25 genes, respectively (Supplementary Table S6). The QTL on scaffold 10/chromosome 4 did not contain the *CYP72A1182* gene but was located 3 Mb away. Functional annotation of genes in this QTL indicates a predominant molecular function of protein binding (GO:0005515), RNA binding (GO:0003723), and mRNA binding (GO:0003729), consistent with proteins involved in regulating other proteins or molecules through selective protein interactions (Hudson and Ortlund, 2014) (Supplementary Fig. S16). The important transcription factor genes that were identified included *DREB2A*, *WRKY*, and *GATA16* within the QTLs. The QTLs also include nine zinc finger proteins, four ubiquitin proteases, three F-box proteins, three ABC transporters, and three cytochrome P450 enzymes.

**Fig. 6.**
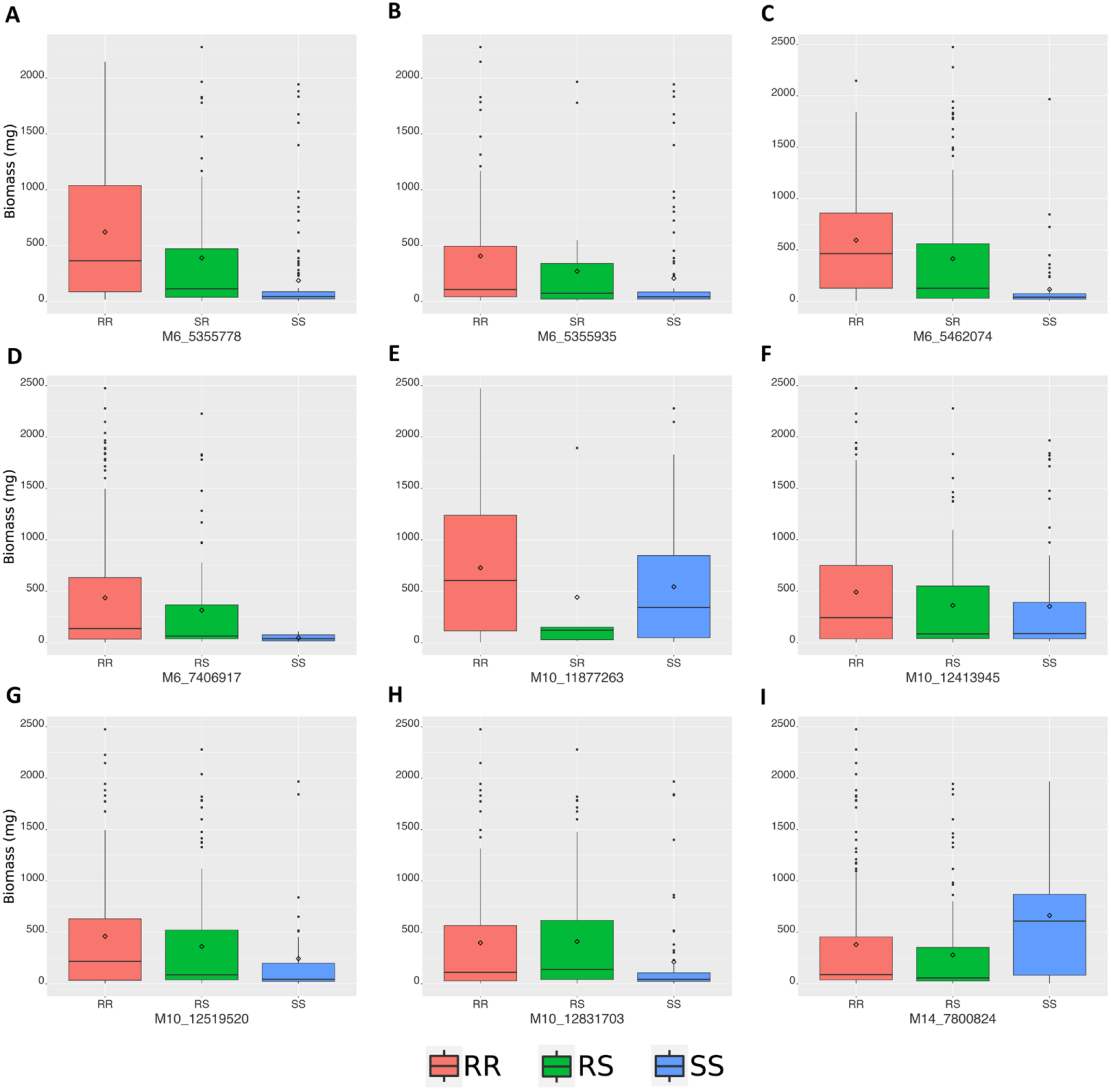
Markers and resistance phenotypes associated with QTLs for tembotrione resistance in *A. palmeri*. (A–D) Markers corresponding to the QTL in scaffold 6 (M6). (E–H) Markers corresponding to the QTL in scaffold 10 (M10). (I) Marker in scaffold 14 (M14). Boxplots represent the range of biomass values after herbicide treatment, including the maximum and minimum values, lower and upper quartiles, and the median. Diamonds indicate the average biomass for each genotype.

For each QTL, multiple markers were identified. In the case of the QTL in scaffold 10, a total of 54 markers were found, while for the QTL in scaffold 6, 94 markers were identified. However, for the QTL in scaffold 14, only a single marker was detected within a 1 Mb range upstream and downstream of the QTL region. The markers associated with scaffolds 10 and 6 displayed a consistent pattern with the evaluated phenotype, where the RR genotype exhibited higher biomasses, while the SS genotype had lower biomasses (Fig. 6). In contrast, the single marker found in scaffold 14 did not demonstrate the same consistency as the markers identified in the other QTLs (Fig. 6I).

## Discussion

The evolution of metabolic herbicide resistance mechanisms in weeds represents a significant challenge in weed control practices (Rigon *et al.*, 2020). These mechanisms enable weeds to metabolize a diverse array of herbicides from various chemical families. Identifying the genes responsible for metabolic resistance and comprehending their regulation holds immense potential for developing improved herbicides and mitigating herbicide resistance. In our current investigation, four consistently up-regulated P450 genes were identified in HPPD-inhibitor resistant *A. palmeri* (Figs 1, 4). These four genes did not have any polymorphisms in the coding sequences between R and S plants. Among these P450s, only one, named CYP72A1182, metabolized the herbicide tembotrione when tested in a heterologous system (Fig. 2). Furthermore, our study revealed that this specific gene is up-regulated in other HPPD-inhibitor-resistant *A. palmeri* populations collected from various fields across the USA, supporting its involvement in conferring herbicide resistance in this globally significant species.

In the past, metabolic herbicide resistance in dicot weeds was relatively uncommon, with most documented cases of metabolic resistance and identified P450 genes occurring in monocots. However, recent studies have reported instances of metabolic HPPD inhibitor resistance in dicots such as *Amaranthus tuberculatus* in 2017 (Kaundun *et al.*, 2017), *A. palmeri* (Küpper *et al.*, 2018) in 2018, and *Raphanus raphanistrum* in 2020 (Lu *et al.*, 2019), making metabolic resistance to HPPD inhibitors the most common metabolic resistance in dicots. Arabidopsis lines expressing *R. raphanistrum* genes *RrCYP704C1* or *RrCYP709B1* were resistant to HPPD inhibitors such as mesotrione (*RrCYP704C1*), tembotrione (*RrCYP709B1*), and isoxaflutole (*RrCYP709B1*) (Lu *et al.*, 2023). In *A. palmeri*, *CYP81CJ2* (referred to as *CYP81E* in the publication) was reported as the primary gene responsible for metabolic resistance in an HPPD-inhibitor-resistant population from Kansas, USA (KCTR) (Aarthy *et al.*, 2022). The population in that study exhibited enhanced metabolism of tembotrione, and *CYP81CJ2* expression, measured by qPCR, was implicated as the likely cause of tembotrione resistance. However, our study indicates that although *CYP81CJ2* is up-regulated in the resistant population, it is not the causal gene for tembotrione resistance in the NER population (Fig. 2). Instead, we have identified *CYP72A1182* as a gene capable of converting tembotrione to hydroxy-tembotrione (Fig. 2). This finding represents the first study that mechanistically validated a gene responsible for herbicide metabolism in *A. palmeri*. Given the complex genetic structure indicated by our QTL mapping study (Figs 5, 6), additional genes may be functionally involved in conferring metabolic resistance to tembotrione, and this question will be the subject of future research.

Metabolic resistance to 2,4-dichlorophenoxyacetic acid (2,4-D) was identified in an *A. tuberculatus* population from Nebraska (de Figueiredo *et al.*, 2022). *CYP81E* has been hypothesized as the gene responsible for 2,4-D metabolic resistance in *A. tuberculatus* (Giacomini *et al.*, 2020). This specific cytochrome P450 gene co-segregated with 2,4-D resistance based on RNA-seq analysis conducted on F_2_ individuals. However, the gene did not co-segregate with HPPD resistance, indicating that the genetic association was specific to 2,4-D resistance. Mechanistic gene validation for function in 2,4-D metabolism was not performed (Giacomini *et al.*, 2020). Interestingly, *CYP81E8* and *CYP81CJ2* alleles from *A. tuberculatus* and *A. palmeri*, respectively, exhibit high sequence similarity, greater than 92%. A similar behavior of *CYP81CJ2* was observed in this study, with higher expression levels in resistant plants of *A. palmeri*; however, when tested in a heterologous system, this enzyme did not metabolize tembotrione (Fig. 2). Based on this finding, we hypothesized that the NER population might have resistance to 2,4-D due to up-regulation of *CYP81CJ2*. The NER population had a resistance index ranging from 2 to 2.5, but complete control was achieved when the recommended field rate of 500 a.e. g ha^−1^ of 2,4-D was applied (data not shown). This suggests that the NER population may indeed be evolving resistance to 2,4-D; however, resistance to tembotrione is not attributed to *CYP81CJ2*, but rather to *CYP72A1182*.

Previous research has highlighted *CYP72A219* as a gene involved in herbicide resistance in weeds. A study indicated that among other metabolic genes, *CYP72A219* had constitutive up-regulation in glufosinate-resistant *A. palmeri* plants, with eight times higher expression levels in resistant plants; however, the study did not perform functional gene validation (Salas-Perez *et al.*, 2018). Furthermore, *BsCYP72A219*, along with *BsCYP81Q32* from *Beckmannia syzigachne*, was up-regulated in mesosulfuron-methyl-resistant plants (Wang *et al.*, 2023). These genes were validated in transgenic rice, where only *CYP81Q32* conferred resistance to ALS inhibitors. Notably, up-regulation of two genes with only one gene having validated metabolic function for herbicide resistance resembled the observed pattern in the present study. Additionally, an alignment of *CYP81CJ2* from *A. palmeri* revealed a 92.7% identity with *CYP81Q32-like* from *Amaranthus tricolor* (XP_057543510.1), a recently sequenced *Amaranthus* species (Wang *et al.*, 2022). This finding indicates a very close relationship between *CYP81CJ2* and *CYP81Q32* and may even represent the same gene, designated with different names. The pattern of co-segregation and up-regulation of these genes in different weed species suggests the presence of common *cis*- or *trans*-elements that potentially regulate expression of multiple P450 genes in resistant plants.

Distinct promoter sequences for *CYP72A1182* were found in R pseudo-F_2_ plants compared with S (Fig. 3), while no such variations were observed for *CYP81CJ2*. QTLs with high effects on the herbicide resistance trait, localized in chromosomes 4, 8, and 15, were also identified (Fig. 5). These QTLs include three cytochrome P450 genes and ABC transporters. While these are important genes and warrant further analysis, a gene responsible for metabolizing tembotrione was identified through RNA-seq analysis and validated, suggesting that the new P450s in the QTL mapping may have a small effect for the resistance mechanism of the NER population. The key findings from the QTL are the diverse set of regulatory genes, including key transcription factors that could play a crucial role in the up-regulation of *CYP72A1182* in resistant plants. Transcription factors such as DREB2A, WRKY, and GATA16, along with the zinc finger family found in the mapping analysis, are known for their involvement in abiotic stress responses (Banerjee and Roychoudhury, 2015; Zhang *et al.*, 2015; Seale, 2020). Significantly, it should be highlighted that the identified QTL on chromosome 4 is located 2–3 Mbp upstream of the *CYP72A1182* gene. This proximity might suggest that the transcription factor within the QTL region may regulate the gene, potentially influencing its expression. This regulatory effect could be particularly relevant due to the presence of a distinct promoter sequence observed in resistant plants.

Promoters of P450s have been investigated in other weed species. Nucleotide polymorphisms were observed in the promoters of *CYP81A12* and *CYP81A21* in the R alleles of multiple herbicide resistant *Echinochloa phyllopogon* plants; however, these single nucleotide polymorphisms did not exhibit a significant association with herbicide resistance (Iwakami *et al.*, 2014). Three SNPs were identified in the promoter region of *BsCYP81Q32* in *B. syzigachne* between the R and S variants, spanning approximately 567–1207 bp upstream of the gene. These SNPs were responsible for increased expression of *GUS* in tobacco leaves transformed with the gene, suggesting the presence of a crucial sequence within that specific region (Wang *et al.*, 2023). Additionally, the researchers identified a transcription factor, *BsTGAL6*, belonging to the bZIP family, which demonstrated efficient binding to the promoter of *BsCYP81Q32* (Wang *et al.*, 2023). This finding suggests the involvement of a *trans*-element that regulates the expression of *BsCYP81Q32* in resistant plants.

Our study successfully identified and validated the metabolic resistant gene, *CYP72A1182*, in the tembotrione-resistant *A. palmeri* population NER. This enzyme showed the ability to metabolize tembotrione into the main metabolite, hydroxy-tembotrione. We have evidence that this gene is highly up-regulated before and/or after herbicide treatment in various resistant *A. palmeri* populations across the USA, when compared with sensitive populations, which poses a potential threat to weed management strategies targeting this species. We also discovered important QTLs associated with the resistance trait, warranting further investigation and validation for *CYP72A1182* regulation. Future research focusing on validating the identified transcription factors will shed light on the regulation of P450 genes and enhance our understanding of their regulation in weed species.

## Supplementary data

The following supplementary data are available at *JXB* online.

Fig. S1. Scheme of crosses performed to obtain the pseudo-F_2_ generation from *Amaranthus palmeri* population HPPD-resistant (NER) × susceptible (NES).

Fig. S2. Gene-wise dispersion estimates.

Fig. S3. Scatterplot of principal components.

Fig. S4. Distance hierarchical clustering heatmap.

Fig. S5. Phylogenetic analysis.

Fig. S6. Relative gene expression of candidate cytochrome P450 genes in parental NES and NER plants.

Fig. S7. Alignment of *CYP81CJ2* and *CYP72A1182* promoter between susceptible and resistant *Amaranthus palmeri*.

Fig. S8. Motifs analysis using MEME-suite tool.

Fig. S9. Motif sequences found in *Amaranthus palmeri CYP72A1182* promoter in sensitive and resistant pseudo-F_2_ plants.

Fig. S10. Dose response of tembotrione in different *Amaranthus palmeri* populations.

Fig. S11. Parental tembotrione and metabolites in HPPD resistant populations of *Amaranthus palmeri* over time after tembotrione application.

Fig. S12. Chemical structures of tembotrione and the main metabolites.

Fig. S13. Representative reverse-phase HPLC chromatogram for *Amaranthus palmeri* populations at 48 HAT with [^14^C]tembotrione.

Fig. S14. Relative gene expression of candidate cytochrome P450 genes in different *Amaranthus palmeri* populations.

Fig. S15. *CYP72A1182* copy number relative to *ALS* in different populations of *Amaranthus palmeri*.

Fig. S16. Functional enrichment of genes from QTLs of scaffold 10, scaffold 6, and scaffold 14.

Table S1. Gene stability assessed by NormFinder algorithm.

Table S2. Primer sequences used for different experiments and their characteristic.

Table S3. Constitutively differentially expressed genes between tembotrione resistant and susceptible *Amaranthus palmeri*.

Table S4. Logistic equation parameters and the resistance index of survival (% of untreated control) for different populations of *Amaranthus palmeri* subjected to different doses of tembotrione.

Table S5. Logistic equation parameters and the resistance index of shoot fresh weight (of untreated control) for different populations of *Amaranthus palmeri* subjected to different doses of tembotrione.

Table S6. Quantitative trait loci found in pseudo-F_2_*Amaranthus palmeri* in cross B and combined cross A + B for the trait of HPPD resistance.

eraf114_suppl_Supplementary_Figures_S1-S16_Tables_S1-S6

## Data Availability

The transcriptome and genome sequencing data that support the findings of this study are available at https://www.ncbi.nlm.nih.gov, with reference numbers GSE243128 and PRJNA1012341, respectively. The gene sequences are available at Genbank, accession numbers OR596705–OR596711.
