# Supplementary material for: Function of cytochrome P450 CYP72A1182 in metabolic herbicide resistance evolution in Amaranthus palmeri populations
Source: J Exp Bot. 2025 Mar 11;76(10):2891–907. doi: 10.1093/jxb/eraf114 (PMC12223498; doi:10.1093/jxb/eraf114)
Supplement: eraf114_suppl_Supplementary_Figures_S1-S16_Tables_S1-S6 [file eraf114_suppl_supplementary_figures_s1-s16_tables_s1-s6.docx]

## Supplementary Data

**CYTOCHROME P450 *CYP72A1182* IS INVOLVED IN EVOLUTION OF METABOLIC RESISTANCE TO TEMBOTRIONE IN *Amaranthus palmeri* POPULATIONS**

Carlos Alberto Gonsiorkiewicz Rigon^1^, Anita Küpper^2^, Crystal Sparks^1^, Jacob Montgomery^1^, Falco Peter^2^, Simon Schepp^2^, Alejandro Perez-Jones^3^, Patrick J. Tranel^4^, Roland Beffa^5^, Franck E. Dayan^1^, Todd A. Gaines^1^

^1^ Colorado State University, Department of Agricultural Biology, Fort Collins, CO 80523, USA

^2^ Bayer AG, Division CropScience, Weed Control Research, 65926 Frankfurt, Germany

^3^ Weed Control Platform Lead, Bayer CropScience, St Louis, MO 63141, USA

^4^ University of Illinois, Department of Crop Sciences, Urbana, IL 61801, USA

^5^ Senior Scientist Consultant, 65835, Liederbach, Germany


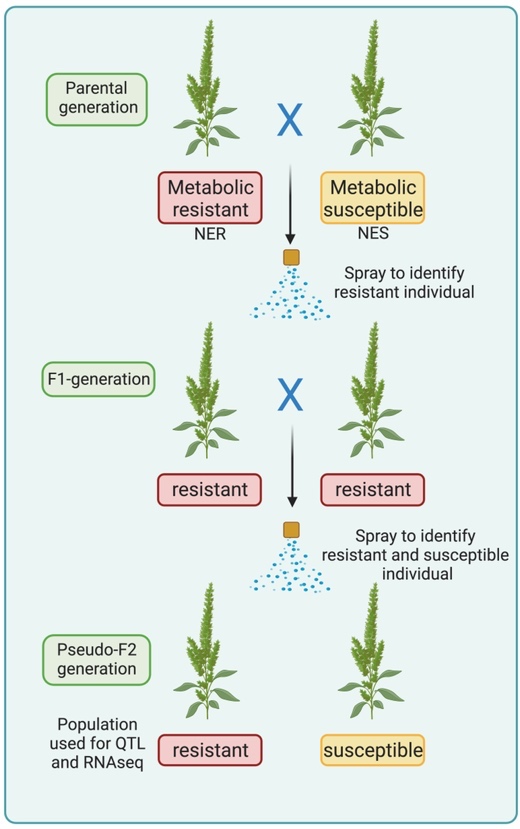


**Supplementary** **Fig. S1.** Scheme of crosses performed to obtain the pseudo-F2 generation from *Amaranthus palmeri* population HPPD-resistant (NER) x susceptible (NES).

**Supplementary** **Fig. S2.** Gene-wise dispersion estimates. The dots indicate every gene expression and its estimates toward the fitted curve. Genes with extremely high dispersion are not shrunk toward the curve.


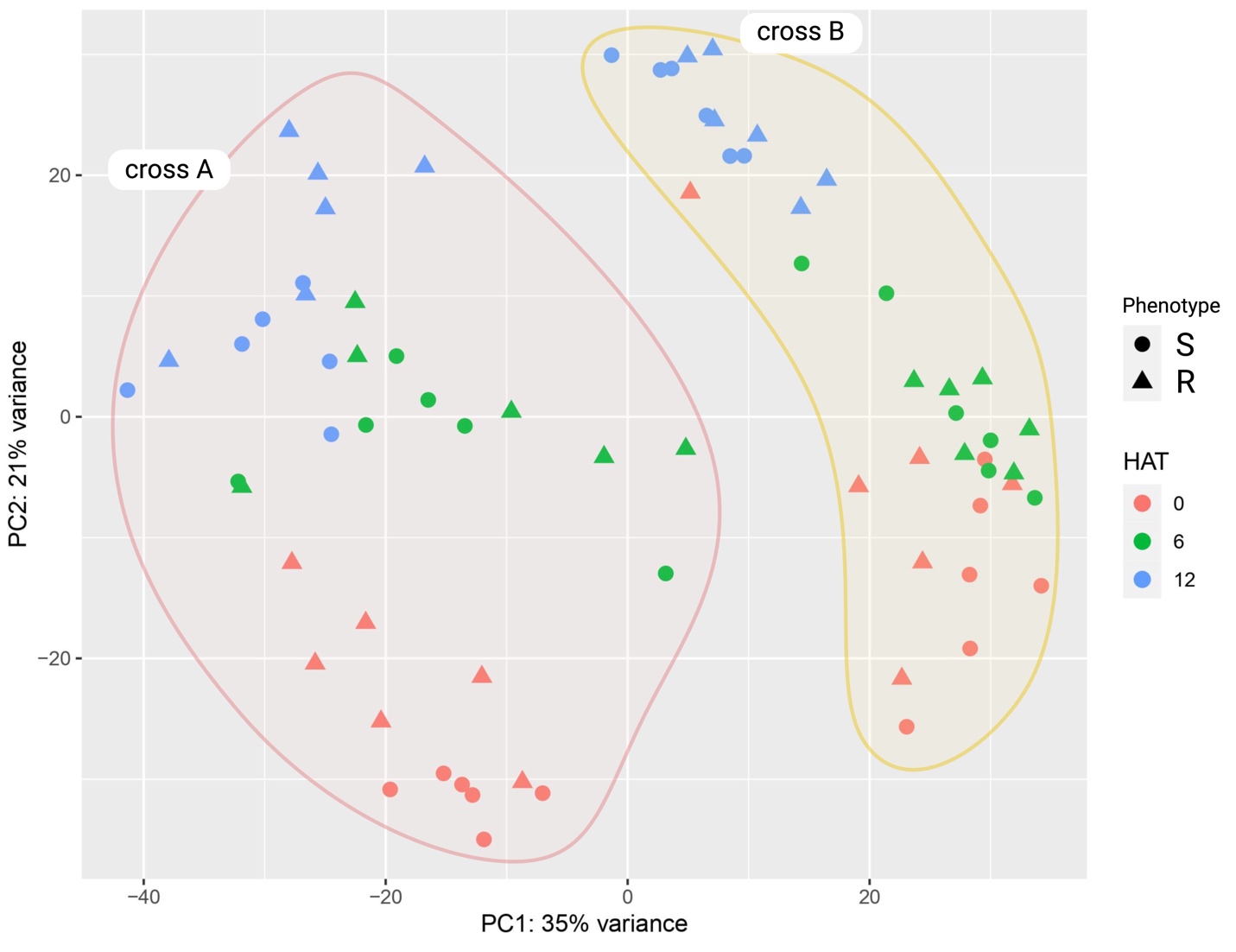
 **Supplementary** **Fig. S3.** Scatterplot of principal components 1 and 2 (35% and 21 % variance explained, respectively) for two different crosses (A and B) between sensitive (S) and HPPD-resistant (R) *Amaranthus palmeri* plants in response to tembotrione before, 6 and 12 HAT.

**Supplementary** **Fig. S4.** Distance hierarchical clustering heatmap of 72 transcriptomes of *Amaranthus palmeri* generated by two crosses (A and B) between sensitive (S) and HPPD-resistant (R) in response to tembotrione application before, 6 and 12 HAT.


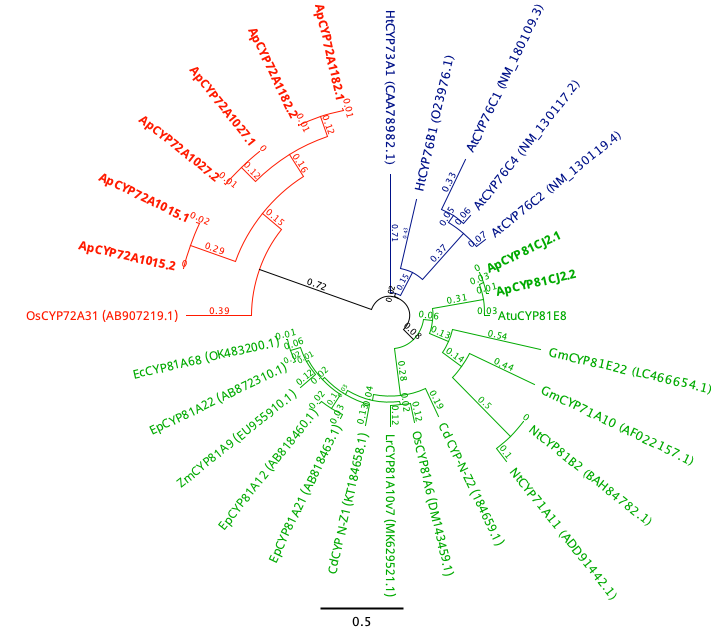


**Supplementary** **Fig. S5.** Phylogenetic analysis of CYP72A1182, CYP72A1027, CYP72A1015 and CYP81CJ2 proteins from *Amaranthus palmeri* and other P450s that metabolize herbicides from different species. Ap – *Amaranthus palmeri*, Atu – *Amaranthus tuberculatus*, At – *Arabidopsis thaliana,* Cd – *Cynodon dactylon*, Ec – *Echinochloa crus-galli*, Ep – *Echinochloa phyllogon*, Gm – *Glycine max*, Ht – *Helianthus tuberorus*, Lr – *Lolium rigidum*, Nt – *Nicotiana tabacum*, Os – *Oryza sativa*, Zm – *Zea mays.*


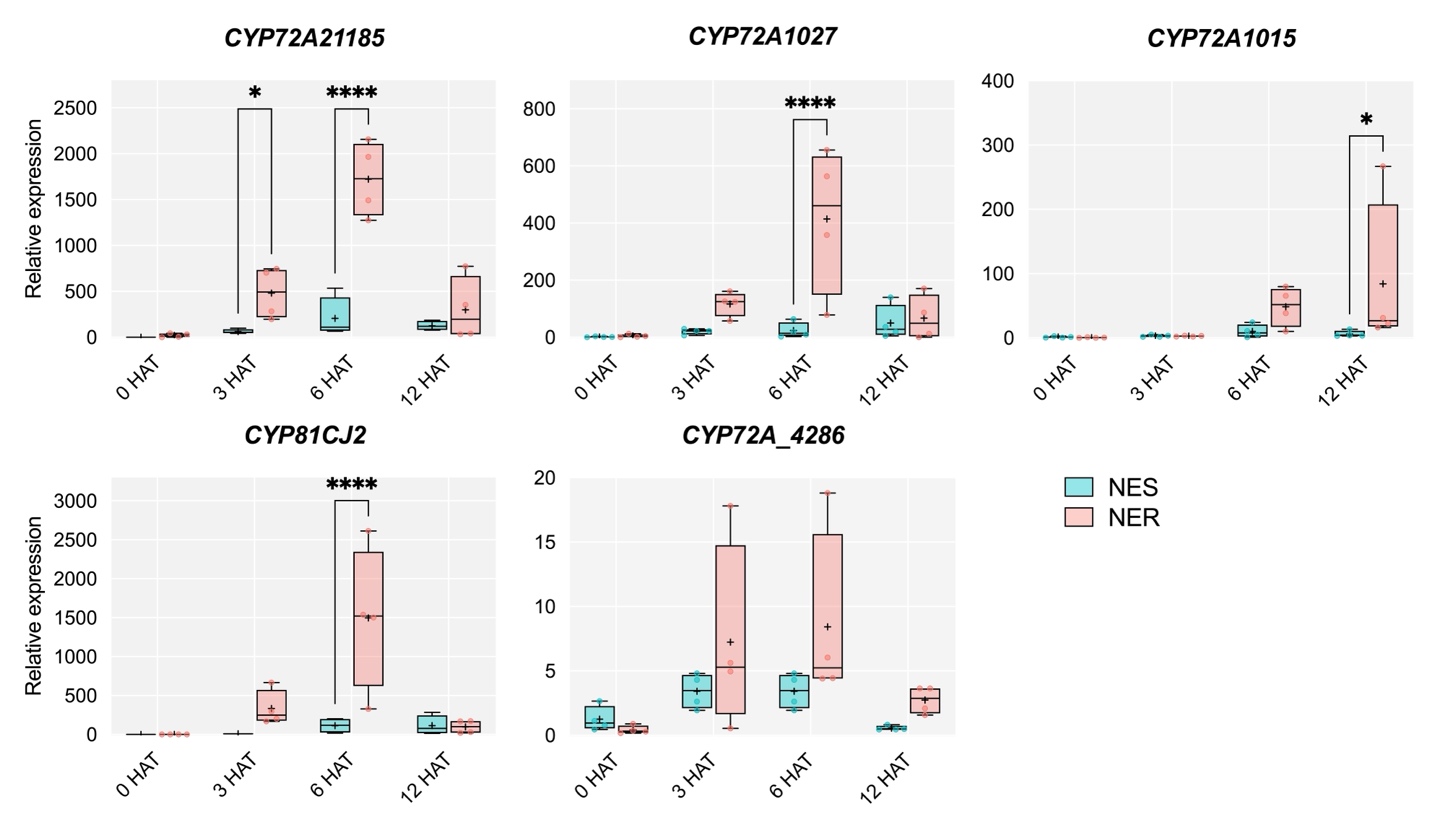


**Supplementary** **Fig. S6.** Relative gene expression of candidate cytochrome P450 genes in parental NES and NER plants. Boxplot indicates the average, the minimum and maximum values obtained from four biological and two technical replicates. Plants samples were collected before, 3, 6 and 12 h after tembotrione application (HAT) (91 g a.i. ha^-1^). The relative expression values were calculated by normalizing to the gene expression of NES untreated, using the average of the normalization gene *18S* and *Actin7*. Statistical differences at each time point were determined using the Fisher's LSD test, with asterisks indicating the significance level: * p < 0.05, and **** p < 0.0001.

**Supplementary** **Fig. S7.** Alignment of *CYP81CJ2* and *CYP72A1182* promoter between susceptible and resistant *Amaranthus palmeri*.

**Alignment of *CYP81CJ2* promoter of S and R plant**

#=======================================

#

# Aligned_sequences: 2

# 1: S1

# 2: R1

# Matrix: EDNAFULL

# Gap_penalty: 10.0

# Extend_penalty: 0.5

#

# Length: 1250

# Identity: 1250/1250 (100.0%)

# Similarity: 1250/1250 (100.0%)

# Gaps: 0/1250 ( 0.0%)

# Score: 6250.0

#

#

#=======================================

S1 1 AAAATACTTCTATAAACTCTTAACACTACCATAAACCAAACCCTACTTTA 50

||||||||||||||||||||||||||||||||||||||||||||||||||

R1 1 AAAATACTTCTATAAACTCTTAACACTACCATAAACCAAACCCTACTTTA 50

S1 51 CCTACATACCACTCACATAAATATTACGATCTATTTTATTAAGTAAAGAA 100

||||||||||||||||||||||||||||||||||||||||||||||||||

R1 51 CCTACATACCACTCACATAAATATTACGATCTATTTTATTAAGTAAAGAA 100

S1 101 TTCTTTGTTTGTTCCTAAAGAAAACTATAAATTAAATACCTATACTCTCT 150

||||||||||||||||||||||||||||||||||||||||||||||||||

R1 101 TTCTTTGTTTGTTCCTAAAGAAAACTATAAATTAAATACCTATACTCTCT 150

S1 151 AAATAACTTTATCTACCATGTGATGGGTGTACCTACGTACATCGGAAAGA 200

||||||||||||||||||||||||||||||||||||||||||||||||||

R1 151 AAATAACTTTATCTACCATGTGATGGGTGTACCTACGTACATCGGAAAGA 200

S1 201 AAATCTTATCTCATTTACCATTCATCTTTCTCAGTTTAACTCAAATTTAT 250

||||||||||||||||||||||||||||||||||||||||||||||||||

R1 201 AAATCTTATCTCATTTACCATTCATCTTTCTCAGTTTAACTCAAATTTAT 250

S1 251 ACCTATTTACAGAAGTAGTTTAACGTGAATATAACTAATAAGTATCCTTA 300

||||||||||||||||||||||||||||||||||||||||||||||||||

R1 251 ACCTATTTACAGAAGTAGTTTAACGTGAATATAACTAATAAGTATCCTTA 300

S1 301 TACACGTTAATTAAATCTAGACAAATACCCGCCCAATTAGTCTCAGACAG 350

||||||||||||||||||||||||||||||||||||||||||||||||||

R1 301 TACACGTTAATTAAATCTAGACAAATACCCGCCCAATTAGTCTCAGACAG 350

S1 351 GTAAATTAATCCTCTTATCCACTTATTTTTATCTTAAAAATTTCATAATT 400

||||||||||||||||||||||||||||||||||||||||||||||||||

R1 351 GTAAATTAATCCTCTTATCCACTTATTTTTATCTTAAAAATTTCATAATT 400

S1 401 AATCAAAAAACTAACGAATTCTAACACATGAAATCACTCCATAACATCCT 450

||||||||||||||||||||||||||||||||||||||||||||||||||

R1 401 AATCAAAAAACTAACGAATTCTAACACATGAAATCACTCCATAACATCCT 450

S1 451 CCACAATACATACATGAAATTAATATCTCATTAATATCCAAAATTATATC 500

||||||||||||||||||||||||||||||||||||||||||||||||||

R1 451 CCACAATACATACATGAAATTAATATCTCATTAATATCCAAAATTATATC 500

S1 501 TTTTCTACTTATTGAAAATCCAAAAACCCTCTATAATATTCATTTAAATA 550

||||||||||||||||||||||||||||||||||||||||||||||||||

R1 501 TTTTCTACTTATTGAAAATCCAAAAACCCTCTATAATATTCATTTAAATA 550

S1 551 CATACTTTCCCATTACCCCGTTTAATATACCTGTTTGATTTTAACATATA 600

||||||||||||||||||||||||||||||||||||||||||||||||||

R1 551 CATACTTTCCCATTACCCCGTTTAATATACCTGTTTGATTTTAACATATA 600

S1 601 TCTCGTTTTAATTTCTCTAGTGAATTAAATCCTCTATAAAAGAGTGCATT 650

||||||||||||||||||||||||||||||||||||||||||||||||||

R1 601 TCTCGTTTTAATTTCTCTAGTGAATTAAATCCTCTATAAAAGAGTGCATT 650

S1 651 TTTAGTACACAATTTTTACACTTACGTTTAGTACAGTAAGAGTATGTGCT 700

||||||||||||||||||||||||||||||||||||||||||||||||||

R1 651 TTTAGTACACAATTTTTACACTTACGTTTAGTACAGTAAGAGTATGTGCT 700

S1 701 AAAGTATAAAAAGTGCATTTTATTAATAATTCCAACAGAAGATTATAGTA 750

||||||||||||||||||||||||||||||||||||||||||||||||||

R1 701 AAAGTATAAAAAGTGCATTTTATTAATAATTCCAACAGAAGATTATAGTA 750

S1 751 TAATTCTTTGATTAATTAATCGGTACTCAAAGTCAAATACCAACATTGAG 800

||||||||||||||||||||||||||||||||||||||||||||||||||

R1 751 TAATTCTTTGATTAATTAATCGGTACTCAAAGTCAAATACCAACATTGAG 800

S1 801 ACTATACAAATATAATAGAGTTTGAATATTTGAGATCAGGTACTATACGA 850

||||||||||||||||||||||||||||||||||||||||||||||||||

R1 801 ACTATACAAATATAATAGAGTTTGAATATTTGAGATCAGGTACTATACGA 850

S1 851 TTATAAGCTAGTTCAAATATATCAAGTTAATATTCCTCAAAAGTATATGA 900

||||||||||||||||||||||||||||||||||||||||||||||||||

R1 851 TTATAAGCTAGTTCAAATATATCAAGTTAATATTCCTCAAAAGTATATGA 900

S1 901 CATAACGAATTAAATCGATGAGAATACTCTCTAGCAGAAAATTTCTATGT 950

||||||||||||||||||||||||||||||||||||||||||||||||||

R1 901 CATAACGAATTAAATCGATGAGAATACTCTCTAGCAGAAAATTTCTATGT 950

S1 951 TAAGTATTTATTATTCAGGTGTAAGAATAAAAGAGAAATTAAACATAATT 1000

||||||||||||||||||||||||||||||||||||||||||||||||||

R1 951 TAAGTATTTATTATTCAGGTGTAAGAATAAAAGAGAAATTAAACATAATT 1000

S1 1001 CACCCGGTAAATCGGGTATTTATATTATAACACAGAGAGCCTCTCTGGCA 1050

||||||||||||||||||||||||||||||||||||||||||||||||||

R1 1001 CACCCGGTAAATCGGGTATTTATATTATAACACAGAGAGCCTCTCTGGCA 1050

S1 1051 GCGAGTGTTGTTAAACACCATAACGATATAATAGTTGTTTCTGTAATTAT 1100

||||||||||||||||||||||||||||||||||||||||||||||||||

R1 1051 GCGAGTGTTGTTAAACACCATAACGATATAATAGTTGTTTCTGTAATTAT 1100

S1 1101 AACTATATCATTATGTTGTCTTAATATTTTTCTCTTGTTGAATTATAAAA 1150

||||||||||||||||||||||||||||||||||||||||||||||||||

R1 1101 AACTATATCATTATGTTGTCTTAATATTTTTCTCTTGTTGAATTATAAAA 1150

S1 1151 TACTTTTTTATACTCTTTATATTTTTTCATTTTTTTTAGTTTATATCAAT 1200

||||||||||||||||||||||||||||||||||||||||||||||||||

R1 1151 TACTTTTTTATACTCTTTATATTTTTTCATTTTTTTTAGTTTATATCAAT 1200

S1 1201 TTTATATTCATCGTTTAAATTATCTTGTCACTTTTTTTAACTTTATTTCA 1250

||||||||||||||||||||||||||||||||||||||||||||||||||

R1 1201 TTTATATTCATCGTTTAAATTATCTTGTCACTTTTTTTAACTTTATTTCA 1250

#---------------------------------------

#---------------------------------------

**Alignment of *CYP72A1182* promoter of S and R plant**

#=======================================

#

# Aligned_sequences: 2

# 1: S1

# 2: R1

# Matrix: EDNAFULL

# Gap_penalty: 10.0

# Extend_penalty: 0.5

#

# Length: 2072

# Identity: 1593/2072 (76.9%)

# Similarity: 1593/2072 (76.9%)

# Gaps: 382/2072 (18.4%)

# Score: 7172.5

#

#

#=======================================

S1 1 AATATATAAAACAAAATAACTCTTTACCTAATAATTAAGGATTA-ATATA 49

|||||||||||.|||||||||||||||||||||||||||||||| |||||

R1 1 AATATATAAAATAAAATAACTCTTTACCTAATAATTAAGGATTACATATA 50

S1 50 TATCTCGAAAACAACATTTACTTCCCTATCCCTATATGTGATACACACCT 99

||||||||||||||||||||||||||||||||| ||||

R1 51 TATCTCGAAAACAACATTTACTTCCCTATCCCT-------------ACCT 87

S1 100 TCACTTTGTTTTCTTCCTTTACTCTCATATATATCCTATTGATTTTTTGT 149

||||||||||||||||||||||||||||||||||||.|||||||||||||

R1 88 TCACTTTGTTTTCTTCCTTTACTCTCATATATATCCGATTGATTTTTTGT 137

S1 150 AGTAGTAGTTGGGTTATAAGGCGAATTTTTGCCCCTACCTTTTCTTGTTG 199

||||||||||.||||||||||||||||||||||.||||||||||||||||

R1 138 AGTAGTAGTTAGGTTATAAGGCGAATTTTTGCCTCTACCTTTTCTTGTTG 187

S1 200 TATATCTTAAAATACTCACTTATTCTCAGTTCTTTTACTACAACTATTAA 249

||||||||||||||||||||||||||||||||||||||||||||||||||

R1 188 TATATCTTAAAATACTCACTTATTCTCAGTTCTTTTACTACAACTATTAA 237

S1 250 CTTCTTATAATACACCTATCTCTTGTTTGGAATGTTATTTATCTTGAGTA 299

||||||||||||||||||||||||||||||||||||||||||||||||||

R1 238 CTTCTTATAATACACCTATCTCTTGTTTGGAATGTTATTTATCTTGAGTA 287

S1 300 GTAGTAGTATAGGTCACATAGGGCGAGTATCTATTTGATACTAGTCTCAA 349

|||||||||| ||||.|||||||||||||||||||||||||

R1 288 GTAGTAGTAT----------GGGCTAGTATCTATTTGATACTAGTCTCAA 327

S1 350 ACCCCTCCCTTCTATC-----TTGAGTATCGGTACATCCTCTCATGCTAG 394

||||||||||| |||| |.||||||.||||||||||||.|||||||

R1 328 ACCCCTCCCTT-TATCTGCCGTCGAGTATGGGTACATCCTCTTATGCTAG 376

S1 395 TCTCTCAGGGGGCCGAGGCATACAAGAAGTTTCACTCTCC---ATGGACG 441

|||||.| ||||||||||.||||||||||||||.|||||| ||.|.|.

R1 377 TCTCTTA-GGGGCCGAGGTATACAAGAAGTTTCTCTCTCCATAATCGTCA 425

S1 442 TTAATAAACTTTCGAACGTTCTGTGTGGGT--------GCAATTGTTACG 483

||||||.|||||||...||||||||||||| ||||||||||||

R1 426 TTAATAGACTTTCGGTTGTTCTGTGTGGGTGCAATTCAGCAATTGTTACG 475

S1 484 TATCATTAGCTAATTGATTTCTATGTGTTGTTTTTCTTATAATGATTATT 533

|||||||||||||||||||||||||||||||||||||||| |||||||||

R1 476 TATCATTAGCTAATTGATTTCTATGTGTTGTTTTTCTTAT-ATGATTATT 524

S1 534 TATCTTGATATTTTCATATAAAACTTGCCTAAGCCGCGTTCCATCAACAT 583

||||||||||||||||||||||||||||||||||||||||||||||||||

R1 525 TATCTTGATATTTTCATATAAAACTTGCCTAAGCCGCGTTCCATCAACAT 574

S1 584 ACTCATCAACAATTGACAGATTTATCGATAATCAACAGCTATCAACAACA 633

||||||||||||||||||||||||||||||||||||||||||||||||||

R1 575 ACTCATCAACAATTGACAGATTTATCGATAATCAACAGCTATCAACAACA 624

S1 634 TTTTAATACCAATAATCTATACATCTAGTGAAAAATTAACACATTTTATA 683

||||||||||||||||||||||||||||||||||||||||||||||||||

R1 625 TTTTAATACCAATAATCTATACATCTAGTGAAAAATTAACACATTTTATA 674

S1 684 ATAATAAACCATTTTCACCGCGAGTTCCATTTACAATGAACAATCTTACA 733

|||||||||||||||||.|||||.||||||||||.|||||||||.|||||

R1 675 ATAATAAACCATTTTCATCGCGAATTCCATTTACGATGAACAATTTTACA 724

S1 734 TTAGAATAACTCTTTTTGTGTTTTAGAGTGTTCTCTGCCAGATATCCTAT 783

|| |||||||||||

R1 725 TT-GAATAACTCTT------------------------------------ 737

S1 784 CTGGTGTTTTAACCCGGTCGGGTAAAGGGAATTGACTAAAAAAATAATAT 833

||||

R1 738 ------------------------------------------AATA---- 741

S1 834 TATAGTTAATATAATACAATATTAGAATTTACATTCCATAATATTGGAGC 883

R1 742 -------------------------------------------------- 741

S1 884 TTATATGATAAGATATTGGGGTTTATATTCAATAATATCGGGGTTTATTA 933

R1 742 -------------------------------------------------- 741

S1 934 AATAATATTGGGGTTTATATGATACAATATAAAACCCGACCCGAAATACC 983

R1 742 -------------------------------------------------- 741

S1 984 TAAGGCAGATATCTTATCTGGCAGAGGGTACTCTGAATGACACTCTTTTT 1033

|||| ||||

R1 742 -----------------------------ACTC-------------TTTT 749

S1 1034 TATATCTCTTTTAAGAGTTTTAGAATAAACCTATGATGAA-TTTTACAAT 1082

||||||||||||||||||||||.||||||..|.||||||| |||||||||

R1 750 TATATCTCTTTTAAGAGTTTTACAATAAATTTTTGATGAATTTTTACAAT 799

S1 1083 GAAATAGCTTTGTACTATAAAAAGAATTCACAAAATCGGAGTAATTTAGT 1132

||||||||.||||||.||||||||||||||||||||||||||||||||||

R1 800 GAAATAGCATTGTACCATAAAAAGAATTCACAAAATCGGAGTAATTTAGT 849

S1 1133 AATTGACAGCAAACCGTAAGCTGTAAATTACTATACTTTCTATAACTTTT 1182

|||.|||||||||||.|||.||.|||||||||||||||.|||||||||||

R1 850 AATCGACAGCAAACCATAACCTATAAATTACTATACTTACTATAACTTTT 899

S1 1183 AAAGA--ATATTAAACCCACCTTTTTTGAGAACTGTTCAAATTACGAGAA 1230

||| | |||||||||||||||||||||||||||.|.|||||||||||||

R1 900 AAA-ATCATATTAAACCCACCTTTTTTGAGAACTTTACAAATTACGAGAA 948

S1 1231 TAAATAAAATGAAGTTAGCAGAATAAAAGAGATGTTTTAAGTAAAAGTGC 1280

||||.|||||||||||||||||.|||||||||||||||||.|||.|||||

R1 949 TAAACAAAATGAAGTTAGCAGAGTAAAAGAGATGTTTTAATTAAGAGTGC 998

S1 1281 GTAATAGTAAATTTTTACTCTGTAATCTATCAACTATTCGTAATGAAATA 1330

|||||||||||.|||||.|||.||||||||||..||||||||||||||||

R1 999 GTAATAGTAAACTTTTATTCTATAATCTATCACGTATTCGTAATGAAATA 1048

S1 1331 TACGTTGTATTCCCATCT-AAAAAAGAAATGATGTGGTTTATAAGTAATA 1379

|||||||||||.|||||| |||||||||||.|||||||||||||||||||

R1 1049 TACGTTGTATTGCCATCTAAAAAAAGAAATAATGTGGTTTATAAGTAATA 1098

S1 1380 CTAAAAATCGGGAAAATAATTTATAAGAAATCTTCCTAAAGAAGCTAATT 1429

||||||||||||||||||||||||||.|.||||||||||||||.||||||

R1 1099 CTAAAAATCGGGAAAATAATTTATAAAACATCTTCCTAAAGAAACTAATT 1148

S1 1430 TTACATTGTAGATCTACTTAATACTAGAGAATATTTAATTAATTATATAT 1479

|||.||||||.|||.|||||||||||||||||||||||||||||||||.|

R1 1149 TTAAATTGTAAATCAACTTAATACTAGAGAATATTTAATTAATTATATCT 1198

S1 1480 AAGTTACATTTAATCCGAAATTTAGTACTACTAAATATGATAAT------ 1523

|||||||||||||||||||||||||||||.||||||||||||||

R1 1199 AAGTTACATTTAATCCGAAATTTAGTACTGCTAAATATGATAATGTACTA 1248

S1 1524 -------AATATTAACAGAATTAAAATGCGATTCAATGATTTAT--TATA 1564

|||||||||.||||||||.|||||||||||||||||| ||||

R1 1249 ATATTTAAATATTAACGGAATTAAAGTGCGATTCAATGATTTATTGTATA 1298

S1 1565 A-------------------------------ATTACTCTATAATGTTAC 1583

| ||||||||||||||||||

R1 1299 AAATAGAGTTTTTTTTCAATGATTTATTGTATATTACTCTATAATGTTAC 1348

S1 1584 TAGCTTATTATTTAACATCTTCTGTACATTTTACTATGATTATGGTTATA 1633

||||||||||||||||||||||||||.||||||||||..|||||||||||

R1 1349 TAGCTTATTATTTAACATCTTCTGTAAATTTTACTATACTTATGGTTATA 1398

S1 1634 TGAAGGAGGCAAAATGATATGAATGATGTAAAATGAAAATGCGTTAAGTT 1683

|||||||.||||..|.|||||||.||||||||||.|||||||||||.|||

R1 1399 TGAAGGAAGCAAGGTCATATGAACGATGTAAAATAAAAATGCGTTAGGTT 1448

S1 1684 ACGCAATAAAGTTAGTAAATATATAACTTAATATGTGTAGATTTTTAATA 1733

|||.||||||||||||||||||.|||.|||||||||..||||||.|||||

R1 1449 ACGTAATAAAGTTAGTAAATATCTAATTTAATATGTACAGATTTATAATA 1498

S1 1734 TTTTTCATTTATACTAATTTCATCCGCTAATCTGAAAAATTTGTTCTTAG 1783

|||||||.||||.|||..|||||||.||||||||..||.||||||||||.

R1 1499 TTTTTCAATTATCCTATATTCATCCACTAATCTGCTAAGTTTGTTCTTAA 1548

S1 1784 GTGAACTGATATAAAAAAGAATGTGTAATCGGTGTATATTTTATTCTAAT 1833

|||||||||||||||||||||||||||||..||||||||||||||..|||

R1 1549 GTGAACTGATATAAAAAAGAATGTGTAATTAGTGTATATTTTATTTGAAT 1598

S1 1834 TTGCTACTTATCATACTTATTATCTTTACATCGTTCATAAAGTCTAACCT 1883

||..|||||||||.|.|||||||.|||||||||.||||||||||| .|||

R1 1599 TTATTACTTATCACAGTTATTATTTTTACATCGCTCATAAAGTCT-GCCT 1647

S1 1884 TCTTAAGAGTCAGAGTAAATAAAACATACTACCTCTTTTTTTCCTACTTT 1933

.|||.| .|.||||||||.|||||||||||||||||||||||||||||||

R1 1648 CCTTCA-TGGCAGAGTAATTAAAACATACTACCTCTTTTTTTCCTACTTT 1696

S1 1934 TAACTTATCATACACCACTAATCATTATTCACCTCTACTAAACCC-AATT 1982

||||||||||||||||||||||||||||||||||||||||||||| ||||

R1 1697 TAACTTATCATACACCACTAATCATTATTCACCTCTACTAAACCCAAATT 1746

S1 1983 CTGTTCTTTACT---------- 1994

||||||||||||

R1 1747 CTGTTCTTTACTATTCTCTGGG 1768

#---------------------------------------

#---------------------------------------


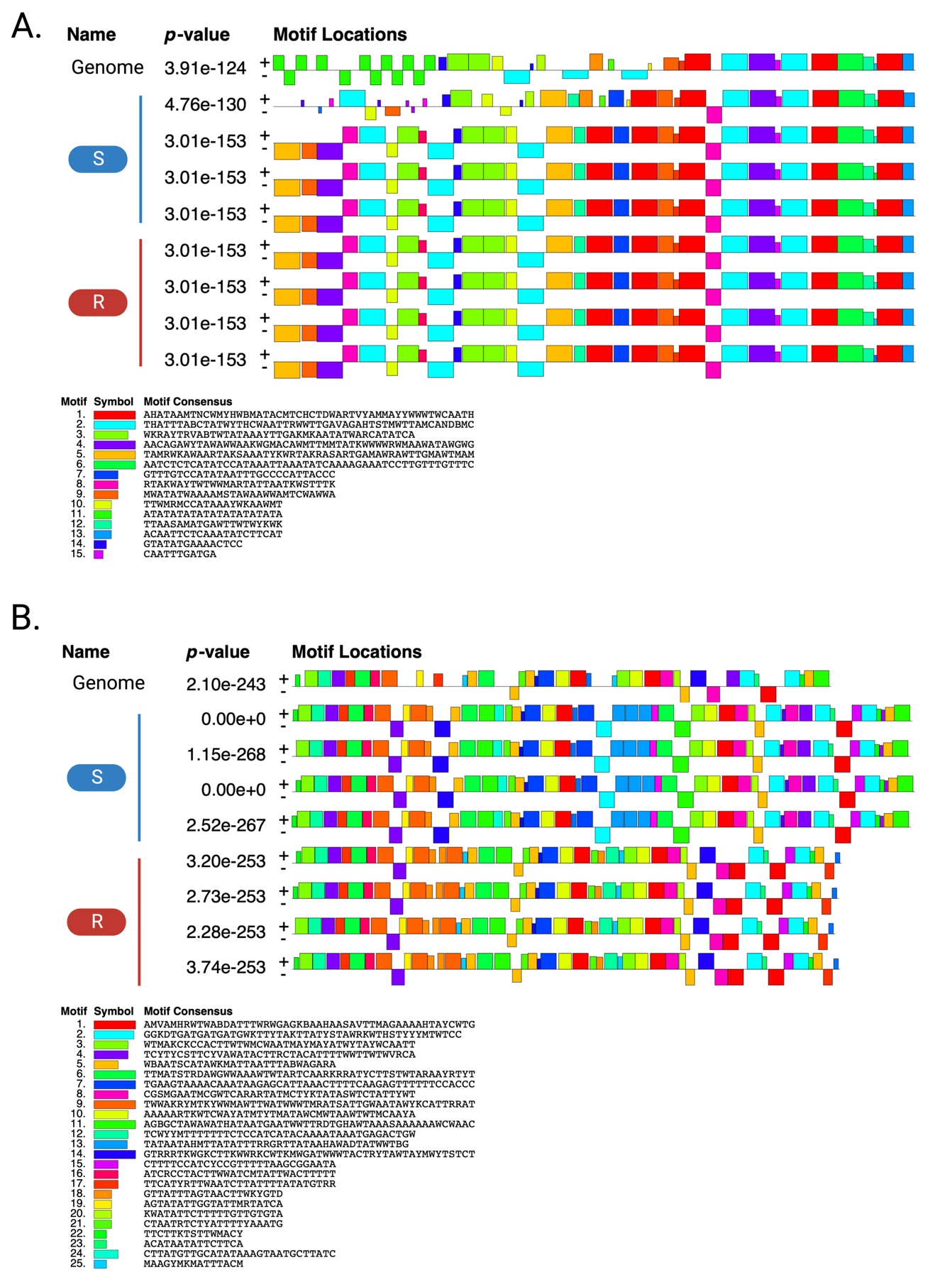


**Supplementary** **Fig. S8.** Motifs analysis using MEME-suite tool (Bailey and Elkan 1994) for *CYP81CJ2* and *CYP72A1182* promoter between S and R samples. Different color indicates different motif.


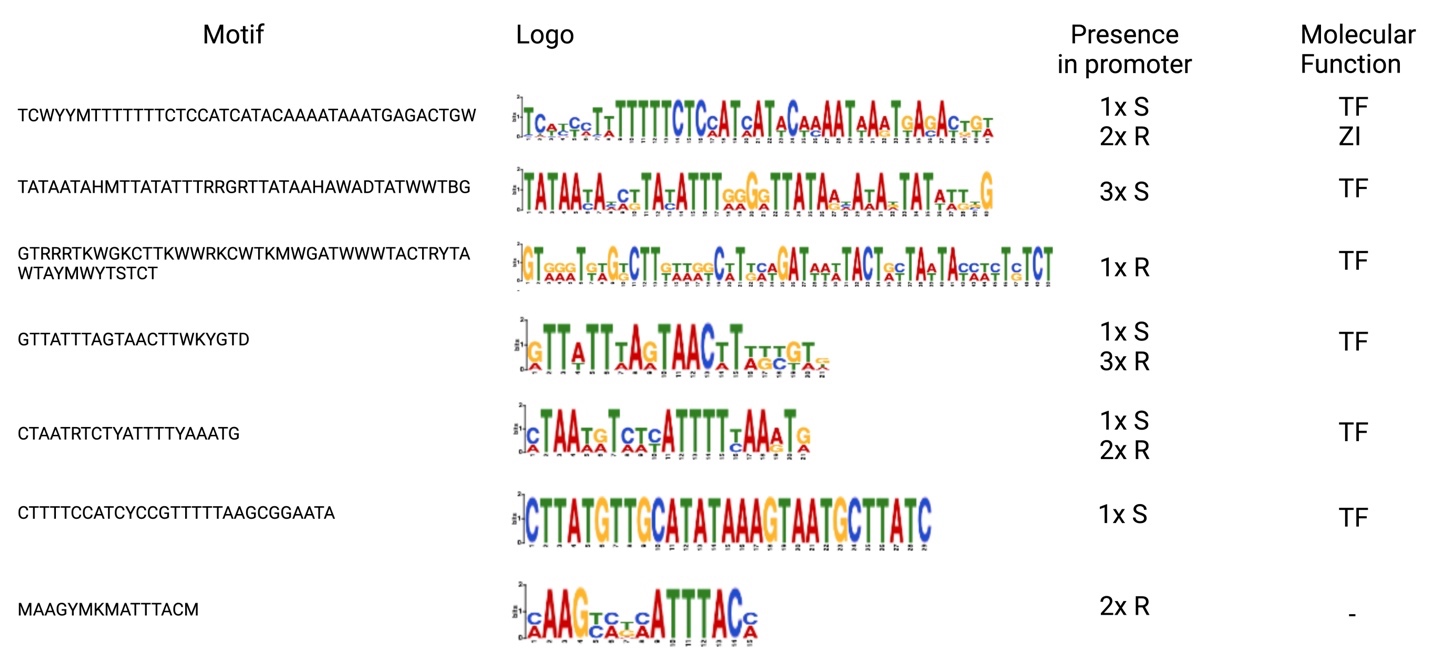


**Supplementary** **Fig. S9.** Motif sequences found in *Amaranthus palmeri* *CYP72A1182* promoter in sensitive and resistant pseudo-F2 plants. TF, transcription factor activity. ZI, zinc ion binding.


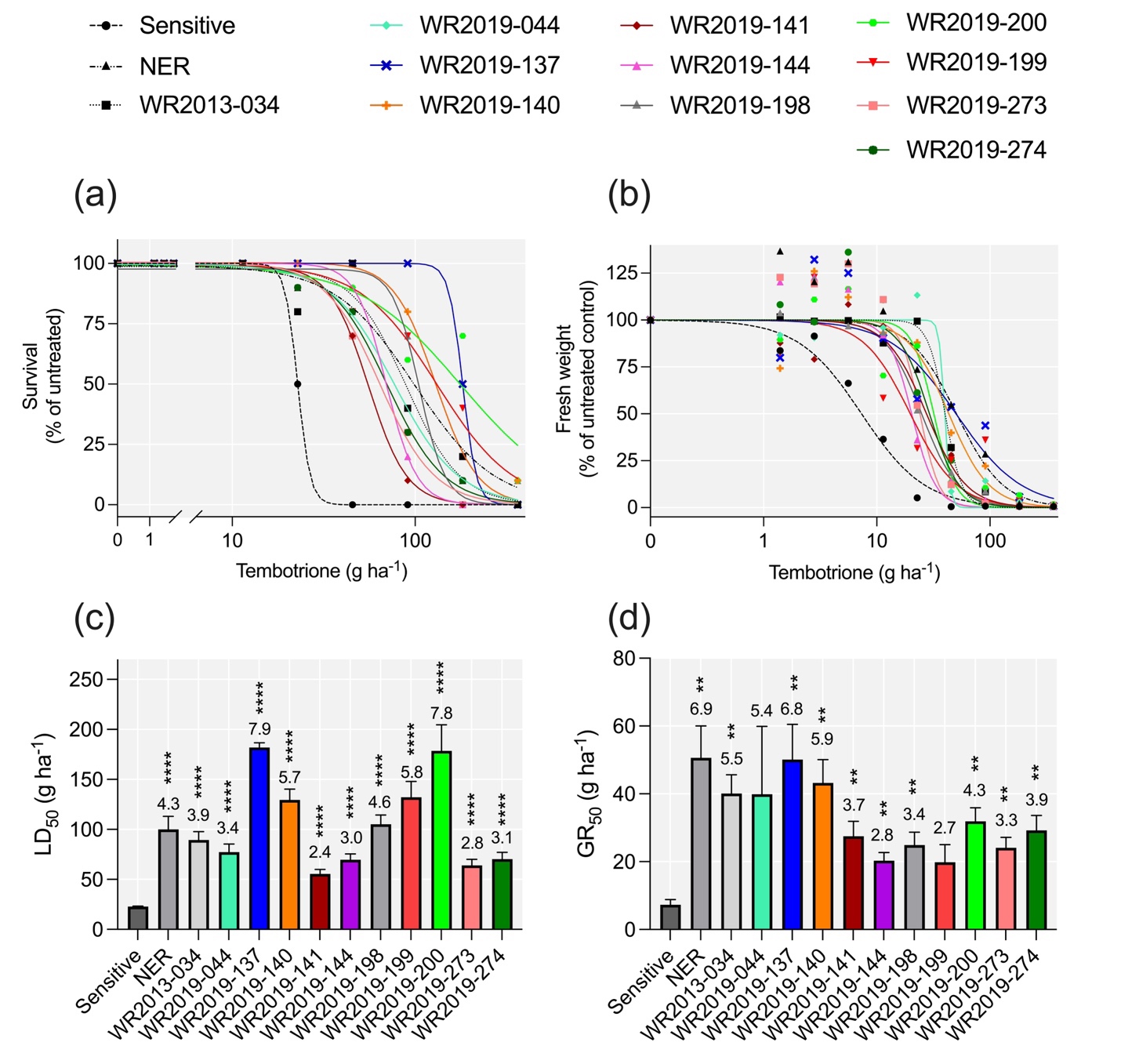


**Supplementary** **Fig. S10.** Dose response of tembotrione in different *Amaranthus palmeri* populations. (a) Survival (% of untreated control) and (b) Fresh shoot weight (% of untreated control) of *A. palmeri* populations submitted to increasing doses of tembotrione. Field rate 91 g a.i. ha^-1^. (c) Estimated LD_50_ and (d) GR_50_ that caused a 50 % reduction in each variable, along with the corresponding resistance index when comparing the parameter with sensitive population. Bars indicates standard error: ** <0.01, ****<0.0001.

**
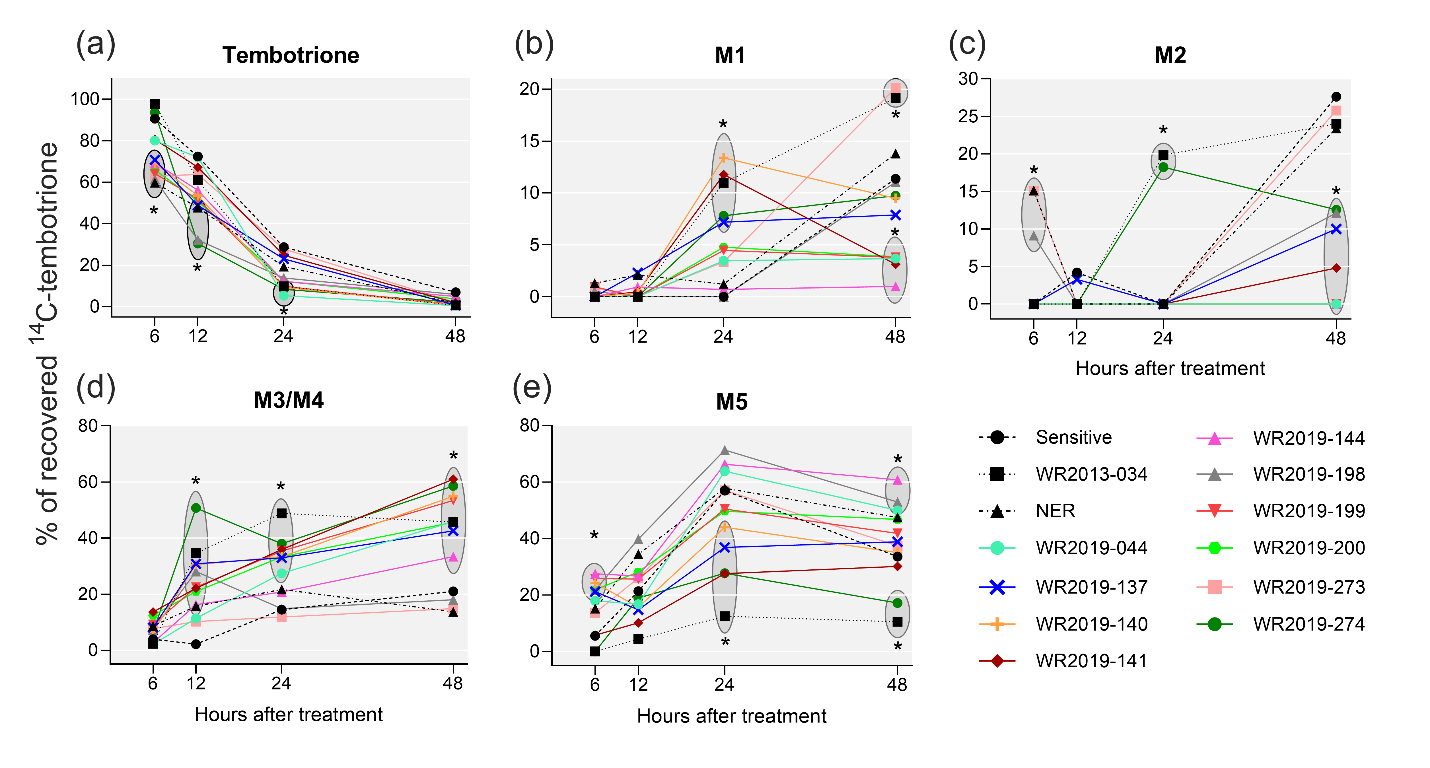
**

**Supplementary** **Fig. S11.** Parental tembotrione and metabolites in HPPD resistant populations of *Amaranthus palmeri* over time after tembotrione application. Average of six plants per population and treatment. (a) Parental tembotrione, (b) Metabolite 1, Glycosylated tembotrione; (c) Metabolite 2; (d) Metabolite 3 and 4 – Hydroxylated-tembotrione. (e) Metabolite 5 – Reduced tembotrione. Asterisk indicates significant differences (p<0.05) between each population with sensitive by Dunnett’s Test. Note: M3 and M4 represent hydroxylated tembotrione, differing only in the position of the hydroxyl group on the left aromatic ring.

**
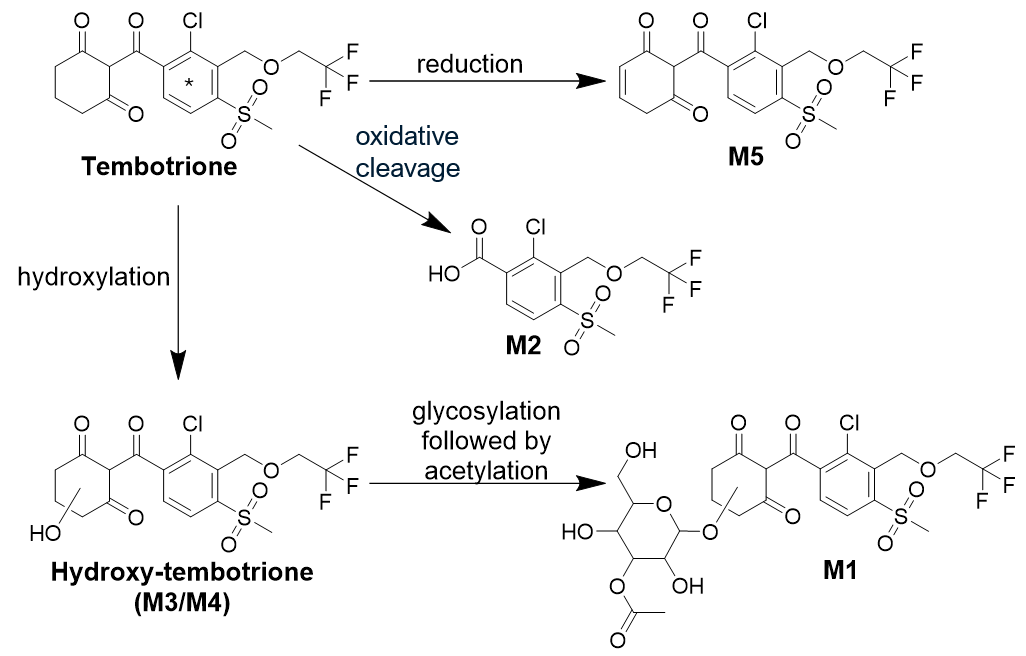
**

**Supplementary** **Fig. S12.** Chemical structures of tembotrione and the main metabolites. Detoxification pathways proposed by Küpper et al. (2018). The asterisk in the tembotrione molecule marks the location of the 14C-label.


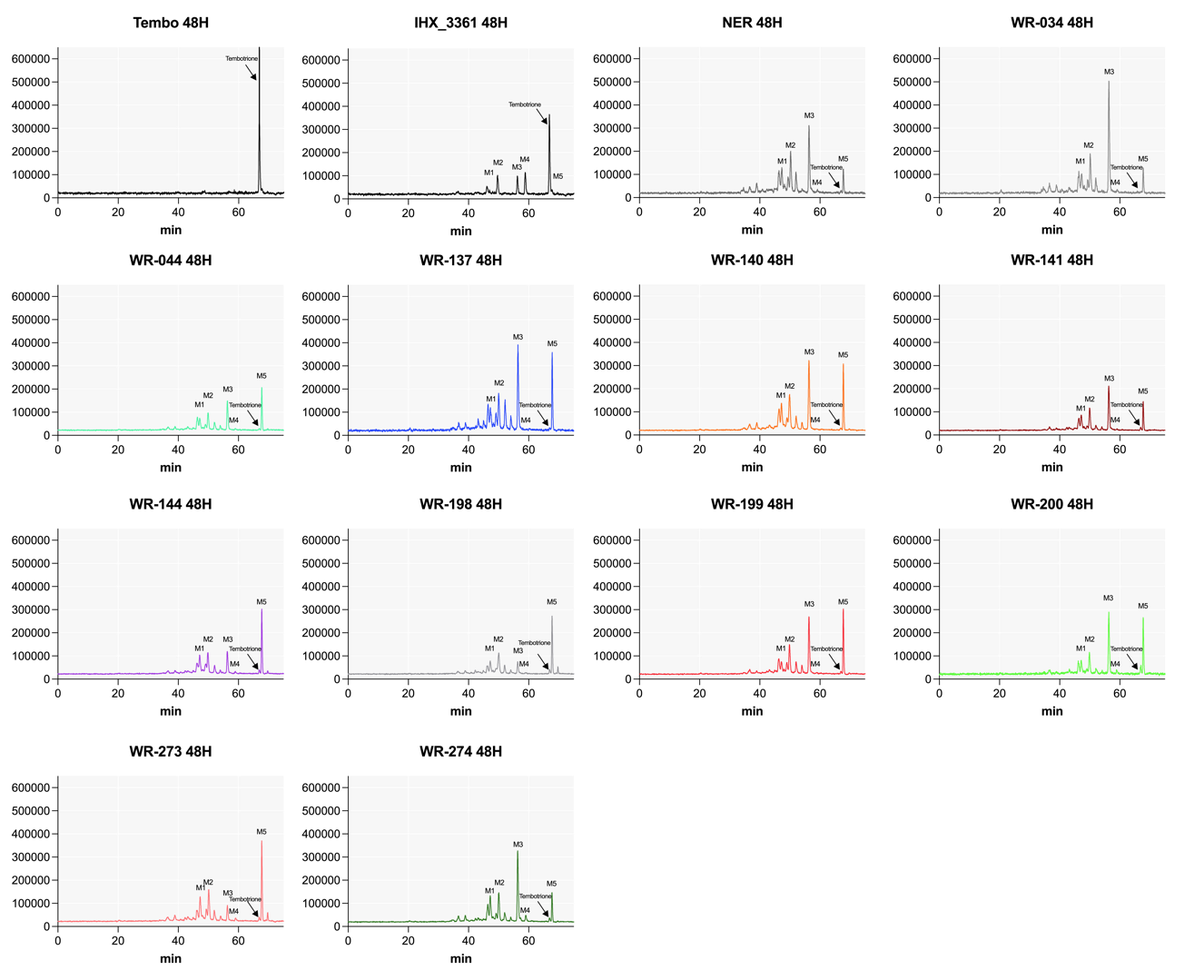


**Supplementary** **Fig. S13.** Representative reverse-phase HPLC chromatogram for *Amaranthus palmeri* populations at 48 HAT with ^14^C-Tembotrione. Retention times of 47.3, 50.1, 56.3, 59.0, 66.9 and 67.7 min correspond to M1, M2, M3, M4, tembotrione and M5, respectively. IHX_3361 is herbicide sensitive control, NER and WR2013-034 are HPPD resistant control.

**
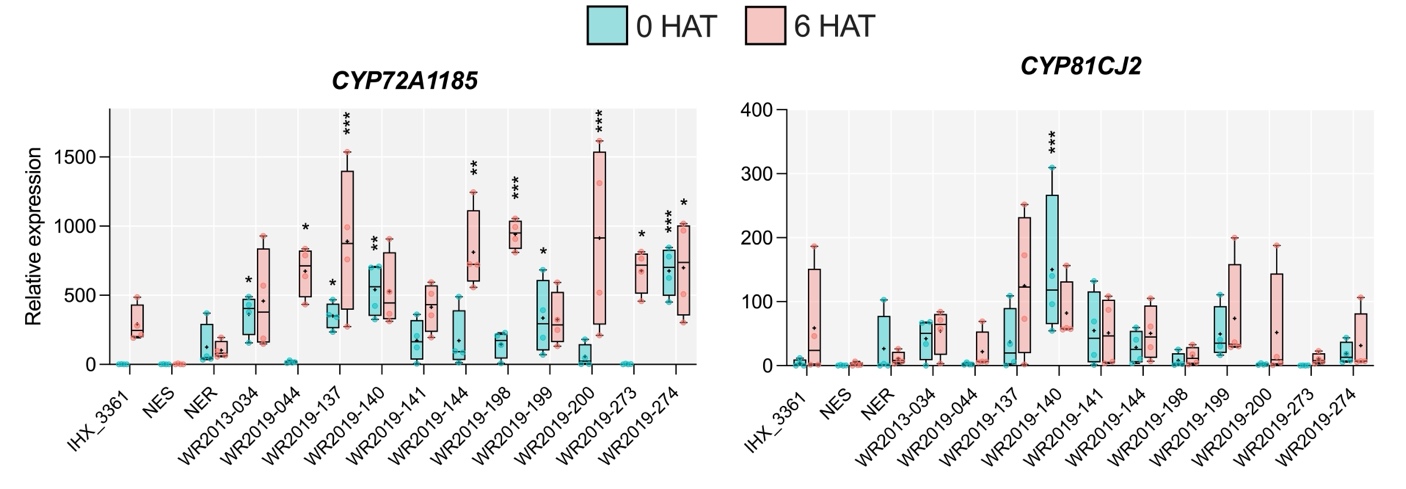
**

**Supplementary** **Fig. S14.** Relative gene expression of candidate cytochrome P450 genes in different *Amaranthus palmeri* populations. Boxplot indicates the average, the minimum and maximum values of four biological and two technical replicates. Plants were collected before (0 HAT) and at 6 h after tembotrione application (HAT) (91 g a.i ha^-1^). Relative expression was calculated using the average of the normalization gene *18S* and normalized relative to the gene expression in the sensitive biotype untreated. IHX_3361 and NES are herbicide sensitive control, NER and WR2013-034 are HPPD resistant control. The asterisk indicates the statistical difference between each population with sensitive for each time point by the Fisher’s LSD test, * <0.05, ** <0.01, *** <0.001.

**
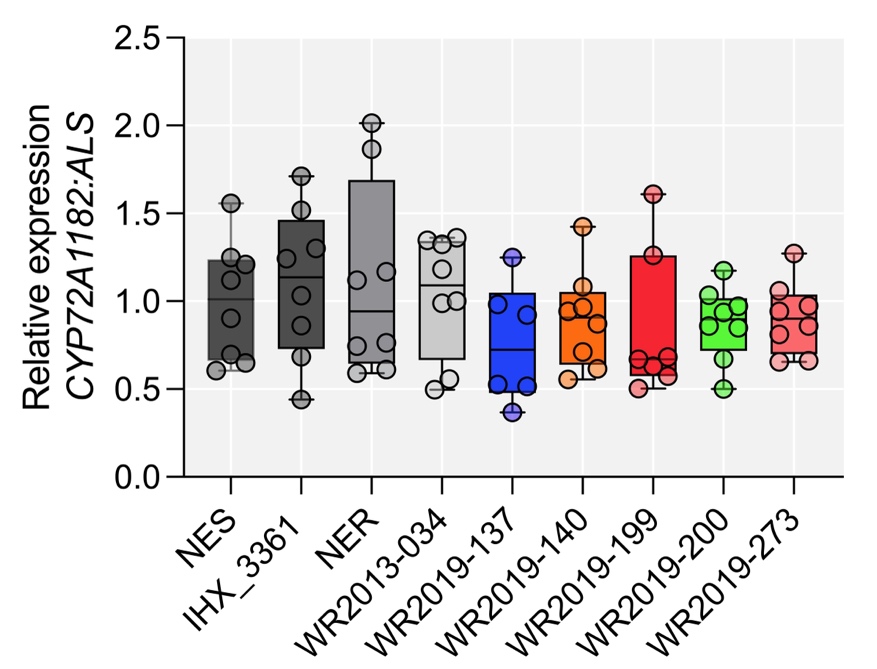
**

**Supplementary** **Fig. S15.** *CYP72A1182* copy number relative to *ALS* in different populations of *Amaranthus palmeri*. NES and IHX_3361 are herbicide sensitive control, NER and WR2013-034 are HPPD resistant control. Circle indicates biological replicate.


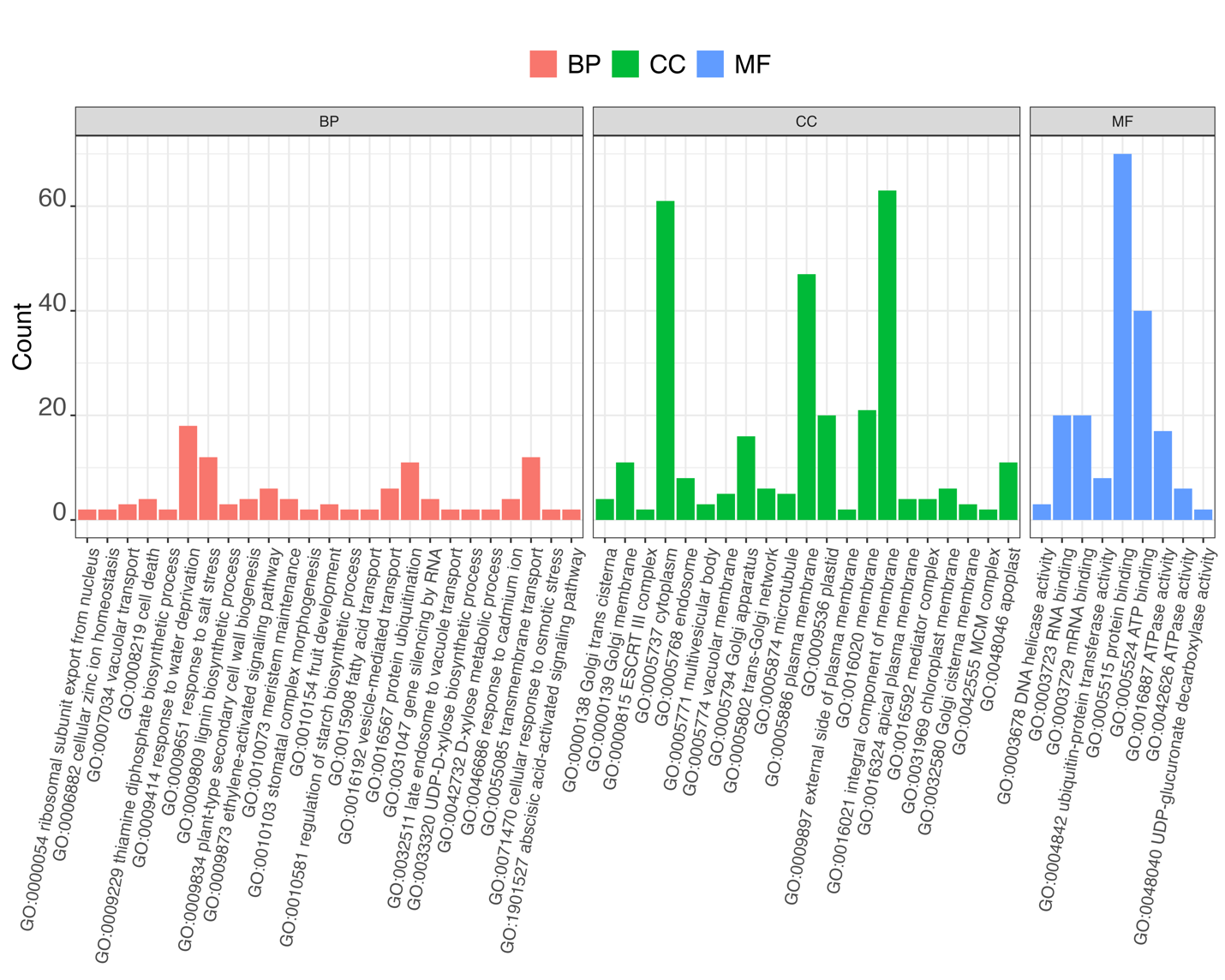


**Supplementary** **Fig. S16.** Functional enrichment of genes from QTLs of scaffold 10, scaffold 6 and scaffold 14. Detailed information of functional annotations of all the genes based on GO classification, including biological process (BP), cellular component (CC) and molecular function (MF).

**Supplementary** **Table S1.** Gene stability assessed by NormFinder algorithm.

| Gene name |  | Stability value |
| --- | --- | --- |
| *18S* |  | 0.110 |
| *ACT* |  | 0.193 |
| *TUB* |  | 0.231 |
| Intergroup variation | NES | NER |
| Group identifier |  |  |
| *18S* | 0.068 | -0.068 |
| *ACT* | -0.149 | 0.149 |
| *TUB* | 0.080 | -0.080 |
| Intragroup variation | NES | NER |
| Group identifier |  |  |
| *18S* | 0.515 | 0.128 |
| *ACT* | 1.048 | 0.746 |
| *TUB* | 1.147 | 1.427 |
| Best gene |  | 18S |
| Stability value |  | 0.110 |
| Best combination of two genes |  | 18S and ACT |
| Stability value for best combination of two genes |  | 0.112 |

NES – Nebraska susceptible, NER – Nebraska HPPD-resistant.

**Supplementary** **Table S2.** Primer sequences used for different experiments and their characteristics.

| **Experiment** | **gene** | **sequence 5' to 3'** | **size (bp)** |
| --- | --- | --- | --- |
| **Gene validation** | qPCR_actin_F | ggctgatgcagaggagattc | 149 |
|  | qPCR_actin_R | gtcccataccaaccatgacacc |  |
|  | 18S rRNA_F | caaccataaacgatgccgacc | 113 |
|  | 18S rRNA_R | cagccttgcgaccatactcc |  |
|  | TUB_F | cattatactgaaggtgctgaac | 211 |
|  | TUB_R | ctttcggagatgggaaaaca |  |
|  | ALSF2 (Gaines et al. 2010) | gctgctgaaggctacgct | 118 |
|  | ALSR2 (Gaines et al. 2010) | gcg ggactgagtcaagaagtg |  |
|  | CYP72A1182_F | gccaaaaaggttagaaaaatgc | 130 |
|  | CYP72A1182_R | taggttgggcattgggttta |  |
|  | CYP72A1027_F | aaagcatcaaaattggcaac | 213 |
|  | CYP72A1027_R | actacgacacctgctggaag |  |
|  | CYP72A219_4286_F | tcgaaagccaaaattgaacc | 158 |
|  | CYP72A219_4286_R | ccttaacactggtgccgaat |  |
|  | CYP72A1015_F | gccggtgtgcaagttaaaat | 232 |
|  | CYP72A1015_R | atgaaaagtgcggcaaaatc |  |
|  | CYP81CJ2_1F | gacaatgatcgcaccaacac | 131 |
|  | CYP81CJ2_1R | cagggaacaatgtggctctt |  |
| **Promoter amplification** | CYP81CJ2_F | ggacaaaggagtgaaatcatcg | 2131 |
|  | CYP81CJ2_R | catgcggaggaaactaccac |  |
|  | CYP72A1182_Pro_F | gggtcactgtcttttgatttaggg | 1951 |
|  | CYP72 A1182_Pro_R | tggtcatccaagctctttca |  |

**Supplementary** **Table S3.** Constitutively differentially expressed genes between tembotrione resistant and susceptible *Amaranthus palmeri*. Adjusted *P*-value <0.01.

| gene ID | log2FC | lfcSE | gene | Molecular function |
| --- | --- | --- | --- | --- |
| MAKER_06269 | 22.6 | 3.43 | Protein of unknown function | - |
| MAKER_31185 | 21.49 | 4.09 | Protein of unknown function | - |
| MAKER_12558 | 5.83 | 0.92 | Disease resistance protein At4g27190-like (LOC104598357) | ADP binding |
| MAKER_03299 | 4.5 | 0.85 | MADS-box transcription factor 23-like | DNA-binding transcription factor activity |
| MAKER_29886 | 4.2 | 0.57 | NADPH--cytochrome P450 reductase (CYP72A219-like) | monooxygenase activity |
| MAKER_32649 | 4.15 | 0.89 | MADS-box transcription factor 27 (MADS27) | DNA-binding transcription factor activity |
| MAKER_08142 | 3.71 | 0.56 | Protein of unknown function | - |
| MAKER_16168 | 3.57 | 0.43 | Glutathione S-transferase U19 (GSTU9) | glutathione transferase activity |
| MAKER_25717 | 3.56 | 0.52 | NADPH--cytochrome P450 reductase (CYP72A219-like) | monooxygenase activity |
| MAKER_15874 | 3.54 | 0.8 | Protein of unknown function | - |
| MAKER_05008 | 3.37 | 0.43 | Scopoletin glucosyltransferase (TOGT1) | scopoletin glucosyltransferase activity |
| MAKER_12487 | 3.29 | 1.53 | Protein of unknown function |  |
| MAKER_12474 | 3.27 | 1.9 | RING-H2 finger protein ATL3 | metal ion binding |
| MAKER_17494 | 3.2 | 1.05 | Protein of unknown function | - |
| MAKER_08144 | 3.19 | 0.4 | glycosyltransferase | - |
| MAKER_34451 | 3.15 | 0.95 | Protein of unknown function | - |
| MAKER_28341 | 3.02 | 0.51 | Vacuolar-processing enzyme beta-isozyme (bVPE) | cysteine-type endopeptidase activity |
| MAKER_21756 | 3.01 | 0.57 | glutathione S-transferase (GST) | transferase activity |
| MAKER_16324 | 2.94 | 0.48 | Protein of unknown function | - |
| MAKER_20644 | 2.93 | 0.54 | UDP-glycosyltransferase 71A15 | transferase activity |
| MAKER_08145 | 2.69 | 0.45 | UDP-glycosyltransferase 71B2-like | UDP-glycosyltransferase activirty |
| MAKER_10107 | 2.64 | 0.44 | Cytochrome P450 81E8 (CYP81E8) | monooxygenase activity |
| MAKER_27594 | 2.61 | 0.78 | Lateral Organ Boundaries (LOB) | DNA binding |
| MAKER_20648 | 2.5 | 0.4 | Betanidin 6-O-glucosyltransferase | glucosyltransferase activity |
| MAKER_21351 | 2.47 | 0.33 | Crocetin glucosyltransferase (GLT2) | glucosyltransferase activity |
| MAKER_25718 | 2.43 | 0.47 | NADPH--cytochrome P450 reductase (CYP72A219-like) | monooxygenase activity |
| MAKER_07960 | 2.38 | 0.39 | Glycosyltransferase GTB type superfamily protein | transferase activity |
| MAKER_04610 | 2.2 | 0.33 | Enhanced pseudomonas susceptibility 1 (EPS1) | acyltransferase activity |
| MAKER_24005 | 2.16 | 0.42 | NADPH:quinone oxidoreductase | NADPH dehydrogenase activity |
| MAKER_16167 | 2.16 | 0.44 | Glutathione S-transferase (GST) | glutathione transferase activity |
| MAKER_06150 | 2.06 | 0.36 | Glutathione S-transferase U19 (GSTU9) | glutathione transferase activity |
| MAKER_04612 | 2.03 | 0.31 | acetyl-transferase-like | transferase activity |
| MAKER_04611 | 2 | 0.34 | BAHD acyltransferase DCR-like protein | transferase activity |
| MAKER_22184 | 2 | 0.46 | Calcium-binding protein (CP1) | binding |
| MAKER_20679 | 1.9 | 0.25 | Detoxification 27 (DTX27) | transmembrane transporter activity |
| MAKER_12408 | 1.81 | 0.37 | Beta-amyrin synthase 1 | intramolecular transferase activity |
| MAKER_12523 | 1.55 | 0.29 | Aldehyde dehydrogenase family 7 member B4 (ALDH7B4) | catalytic activity |
| MAKER_11325 | -1.49 | 5.1123 | Protein of unknown function | - |
| MAKER_11763 | -2.55 | 0.4656 | Abscisic-aldehyde oxidase (AAO3) | aldehyde oxireductase |
| MAKER_29818 | -3.04 | 1.335 | Abscisic acid receptor (PYL4) | abscisic acid binding |

**Supplementary** **Table S4.** Logistic equation parameters and the resistance index (RI) of survival (% of untreated control) for different populations of *Amaranthus palmeri* subjected to different doses of tembotrione.

| populations | b | d | LD_50_ | | | | RI | *p*-value |
| --- | --- | --- | --- | --- | --- | --- | --- | --- |
|  |  |  | dose  (g ha^-1^) | lower CI (95%) | upper CI (95%) | SE |  |  |
| Sensitive | 12.6 | 100.0 | 22.8 | 21.8 | 23.8 | 0.5 | - |  |
| NER | 1.9 | 98.8 | 99.9 | 74.5 | 125.5 | 12.9 | 4.3 | <0.001 |
| WR2013-034 | 2.9 | 98.5 | 89.4 | 73.1 | 105.8 | 8.3 | 3.9 | <0.001 |
| W2019-044 | 2.5 | 99.5 | 77.3 | 61.6 | 93.0 | 8.0 | 3.4 | <0.001 |
| W2019-137 | 11.6 | 100.0 | 181.9 | 172.8 | 191.1 | 4.6 | 7.9 | <0.001 |
| W2019-140 | 3.8 | 100.3 | 129.4 | 108.2 | 150.6 | 10.8 | 5.7 | <0.001 |
| W2019-141 | 4.1 | 98.9 | 55.4 | 46.8 | 64.5 | 4.4 | 2.4 | <0.001 |
| W2019-144 | 5.2 | 100.0 | 69.6 | 57.8 | 81.3 | 5.9 | 3.0 | <0.001 |
| W2019-198 | 6.2 | 97.3 | 105.2 | 87.2 | 123.24 | 9.1 | 4.6 | <0.001 |
| W2019-199 | 2.1 | 99.5 | 132.1 | 100.4 | 163.2 | 15.8 | 5.8 | <0.001 |
| W2019-200 | 1.5 | 99.0 | 178.3 | 127.6 | 229.6 | 25.9 | 7.8 | <0.001 |
| W2019-273 | 2.8 | 100.6 | 63.8 | 51.9 | 75.9 | 6.1 | 2.8 | <0.001 |
| W2019-274 | 2.8 | 99.5 | 70.3 | 57.1 | 83.6 | 6.7 | 3.1 | <0.001 |

b: slope; d: upper limit; LD_50_: herbicide dose that causes a 50% reduction in survival, CI: confidence interval of the parameter LD_50_ (α = 0.05); and RI: resistance index = LD_50_ ratio between respective population and sensitive.

**Supplementary** **Table S5.** Logistic equation parameters and the resistance index (RI) of shoot fresh weight (of untreated control) for different populations of *Amaranthus palmeri* subjected to different doses of tembotrione.

| Populations | b | d | GR50 | | | | RI | *p*-value |
| --- | --- | --- | --- | --- | --- | --- | --- | --- |
|  |  |  | dose  (g ha^-1^) | lower CI (95%) | upper CI (95%) | SE |  |  |
| Sensitive | 1.5 | 100 | 7.3 | 4.3 | 10.4 | 1.5 | - | - |
| NER | 2.1 | 100 | 50.6 | 32.2 | 69.0 | 9.3 | 6.9 | <0.01 |
| WR2013-034 | 5.7 | 100 | 40.1 | 29.2 | 51.1 | 5.5 | 5.5 | <0.01 |
| W2019-044 | 17.9 | 100 | 39.9 | 0.65 | 79.1 | 19.9 | 5.4 | 0.1 |
| W2019-137 | 1.5 | 100 | 50.2 | 29.6 | 70.6 | 10.4 | 6.8 | <0.01 |
| W2019-140 | 2.4 | 100 | 43.2 | 29.6 | 70.6 | 6.9 | 5.9 | <0.01 |
| W2019-141 | 2.5 | 100 | 27.5 | 18.9 | 36.1 | 4.4 | 3.7 | <0.01 |
| W2019-144 | 4.1 | 100 | 20.3 | 15.6 | 25.0 | 2.4 | 2.8 | <0.01 |
| W2019-198 | 2.6 | 100 | 24.9 | 17.4 | 32.4 | 3.8 | 3.4 | <0.01 |
| W2019-199 | 2.1 | 100 | 19.8 | 9.5 | 30.0 | 5.2 | 2.7 | 0.06 |
| W2019-200 | 4.5 | 100 | 31.9 | 23.9 | 39.8 | 4.0 | 4.3 | <0.01 |
| W2019-273 | 5.1 | 100 | 24.1 | 17.8 | 30.2 | 3.1 | 3.3 | <0.01 |
| W2019-274 | 3.3 | 100 | 29.2 | 20.6 | 37.8 | 4.4 | 3.9 | <0.01 |

b: slope; d: upper limit; GR_50_: herbicide dose that causes a 50% reduction in shoot fresh weight, CI: confidence interval of the parameter GR_50_ (α = 0.05); and RI: resistance index = GR_50_ ratio between respective population and sensitive.

**Supplementary** **Table S6.** Quantitative trait loci found in pseudo-F2 *Amaranthus palmeri* in cross B and combined cross A + B for the trait of HPPD resistance. Position are based on available genome draft of *A. palmeri* (Montgomery et al. 2020).

| QTL | LOD score | Position | Interval | | Chromosome | Annotated genes | PVE |
| --- | --- | --- | --- | --- | --- | --- | --- |
| **Cross B** | | | | | | |  |
| Scaffold_10 | 11.6 | 11877263 | 11865155 | 11890395 | 4 | 77 | 23 % |
|  | 8.4 | 10336852 | 10224431 | 10336879 |  |  |  |
|  | 7.9 | 10337656 | 10336879 | 10482794 |  |  |  |
| Scaffold_81 | 10.1 | 19632215 | 18232833 | 20375576 | 2 | 78 | 20 % |
|  | 8.6 | 18232833 | 18186955 | 18238951 |  |  |  |
| Scaffold_6 | 9.5 | 16851238 | 15842545 | 16851238 | 8 | 7 | 19 % |
|  | 8.7 | 15843735 | 15752153 | 15907859 |  |  |  |
| Scaffold_14 | 8.0 | 7800824 | 2267056 | 7800824 | 15 | 50 | 16 % |
|  | 7.9 | 4777664 | 4754252 | 4917017 |  |  |  |
|  | 7.9 | 2267056 | 2226097 | 2271946 |  |  |  |
| **combined cross A + B** | | | | | | |  |
| Scaffold_10 | 12.25 | 11877263 | 10877263 | 12877263 | 4 | 105 | 15 % |
|  | 7.84 | 11332443 |  |  |  |  |  |
| Scaffold_6 | 7.81 | 5462074 | 4962074 | 5962074 | 8 | 32 | 10 % |
|  | 7.60 | 16851238 | 15150809 | 17351238 |  | 164 |  |
|  | 7.00 | 15650809 |  |  |  |  |  |
|  | 6.68 | 15617367 |  |  |  |  |  |
| Scaffold_14 | 7.87 | 7800824 | 7300824 | 8300824 | 15 | 25 | 10 % |

PVE – phenotype variation explained.

**References**

Bailey T. L., and C. Elkan. (1994) Fitting a mixture model by expectation maximization to discover motifs in biopolymers. Proc Int Conf Intell Syst Mol Biol 2:28-36.

Gaines T. A., W. Zhang, D. Wang, B. Bukun, S. T. Chisholm, D. L. Shaner, S. J. Nissen, W. L. Patzoldt, P. J. Tranel, A. S. Culpepper, T. L. Grey, T. M. Webster, W. K. Vencill, R. D. Sammons, J. Jiang, C. Preston, J. E. Leach, and P. Westra. (2010) Gene amplification confers glyphosate resistance in *Amaranthus palmeri*. Proceedings of the National Academy of Sciences, USA 107:1029-1034.

Montgomery J. S., D. Giacomini, B. Waithaka, C. Lanz, B. P. Murphy, R. Campe, J. Lerchl, A. Landes, F. Gatzmann, A. Janssen, R. Antonise, E. Patterson, D. Weigel, and P. J. Tranel. (2020) Draft genomes of *Amaranthus tuberculatus*, *Amaranthus hybridus*, and *Amaranthus palmeri*. Genome Biol. Evol. 12:1988-1993.
